# The effect of *Aspalathus linearis* (Burm.f.) R.Dahlgren and its compounds on tyrosinase and melanogenesis

**DOI:** 10.1038/s41598-021-86410-z

**Published:** 2021-03-29

**Authors:** Analike Blom van Staden, Carel B. Oosthuizen, Namrita Lall

**Affiliations:** 1 Department of Plant and Soil Sciences, University of Pretoria, Pretoria, South Africa; 2 School of Natural Resources, University of Missouri, Columbia, USA; 3 College of Pharmacy, JSS Academy of Higher Education and Research, Mysore, India

**Keywords:** Cell biology, Computational biology and bioinformatics, Enzymes, Natural products

## Abstract

Pigmentation, a process controlled by melanogenesis, plays a vital role in protecting the skin against harmful ultraviolet rays. The level of protection is compromised in case of hypopigmentation. This study aimed to evaluate an *Aspalathus linearis* extract, fractions and phytoconstituents, for their efficacy on melanogenesis stimulation. Fifteen compounds were kinetically assessed against tyrosinase; the rate-limiting enzyme of melanogenesis. Aspalathin and catechin significantly (*p* value < 0.001) increased the enzymatic rate, showing 50% stimulatory effects at 119.70 ± 2.06 µg/mL and 143.30 ± 2.74 µg/mL, respectively, by acting as subversive substrates. Five compounds inhibited the enzyme’s activity, of which four exhibited competitive inhibition. To investigate the molecular interactions between the compounds and the active site, molecular docking was done, using tyrosinase (PBD: 2Y9X) and tyrosinase related protein 1 (PBD: 5M8P). All the compounds docked successfully with acceptable docking scores. Further quantitative structure–activity relationship analysis identified potential functional groups, linked to the specific activity. The crude extract, its fractions, and compounds exhibited low antiproliferative activity with 50% cell viability at concentrations higher than 100 µg/mL. Finally, both aspalathin and catechin exhibited a significant increase (4.5%) in melanin production at 119.82 µg/mL and 76.92 µg/mL, respectively. This is the first report of *A. linearis’* compounds on skin re-pigmentation.

## Introduction

*Aspalathus linearis* (Burm.f.) R.Dahlgren, commonly known as Rooibos, has been reported for numerous bioactivities; however, investigations regarding the effect of Rooibos on melanogenesis are lacking^1–5^. Previously, it was reported that Rooibos does not have an inhibitory effect on tyrosinase; however, it was not determined whether it could potentially increase the enzyme's activity^6^. Furthermore, there are no reports on the effect of aspalathin, a major constituent in Rooibos, on the melanogenesis pathway^7^. Tyrosinase regulates the rate-limiting step in the melanogenesis pathway and plays a vital role in skin pigmentation^8^. Tyrosinase catalyses the conversion of L-tyrosine to L-dopaquinone, which spontaneously converts into L-dopachrome^9,10^. This conversion results in the production of a product that can be quantified by measuring the absorbance at 492 nm^11^. The oxidation of L-tyrosine to L-dopaquinone is a lengthy step resulting in a lag phase in the kinetic curve; thereafter, a steep gradient is observed for the formation of L-dopaquinone and L-dopachrome until equilibrium is reached^12^. The tyrosinase active site contains two Cu^2+^ ions, which are crucial for enzymatic activity and promotes pigmentation^9^. The amount of pigmentation is dependent on the availability and accessibility of the enzyme’s active site, which could be influenced by an inhibitor^13^. In addition, subversive substrate activity could result in an increase in enzymatic rate. It has been reported that it is not the number of tyrosinase enzyme present within the cell that influences pigmentation, but rather the rate of the enzyme activity^8^. Tyrosinase is an oxidising agent resulting in the exchange of electrons^14^. Therefore, the number of hydroxy and carbonyl groups plays an important role in the interaction of the compounds with the active site residues. The binding poses and potential affinity of ligands to the active site and the type of interactions involved can be identified through computational modelling.

The present study aimed to investigate the intervention of *A. linearis*, its fractions and major constituents with tyrosinase and melanogenesis. In addition, molecular docking studies of the phytochemicals were conducted, and quantitative structure–activity relationship (QSAR) models were used to support the tyrosinase-related activity. R-group analysis was utilised to explain the interactions of the compounds with the enzyme. Finally, the effect of the samples on melanogenesis were further investigated through in vitro analysis on human melanocytes. The results obtained in this study provides the potential mechanism of how *A. linearis* affects melanogenesis.

## Results and discussion

### Quantification of pure compounds using ultra-performance liquid chromatography: quantitative time of flight (UPLC-QToF)

**Figure 1:**
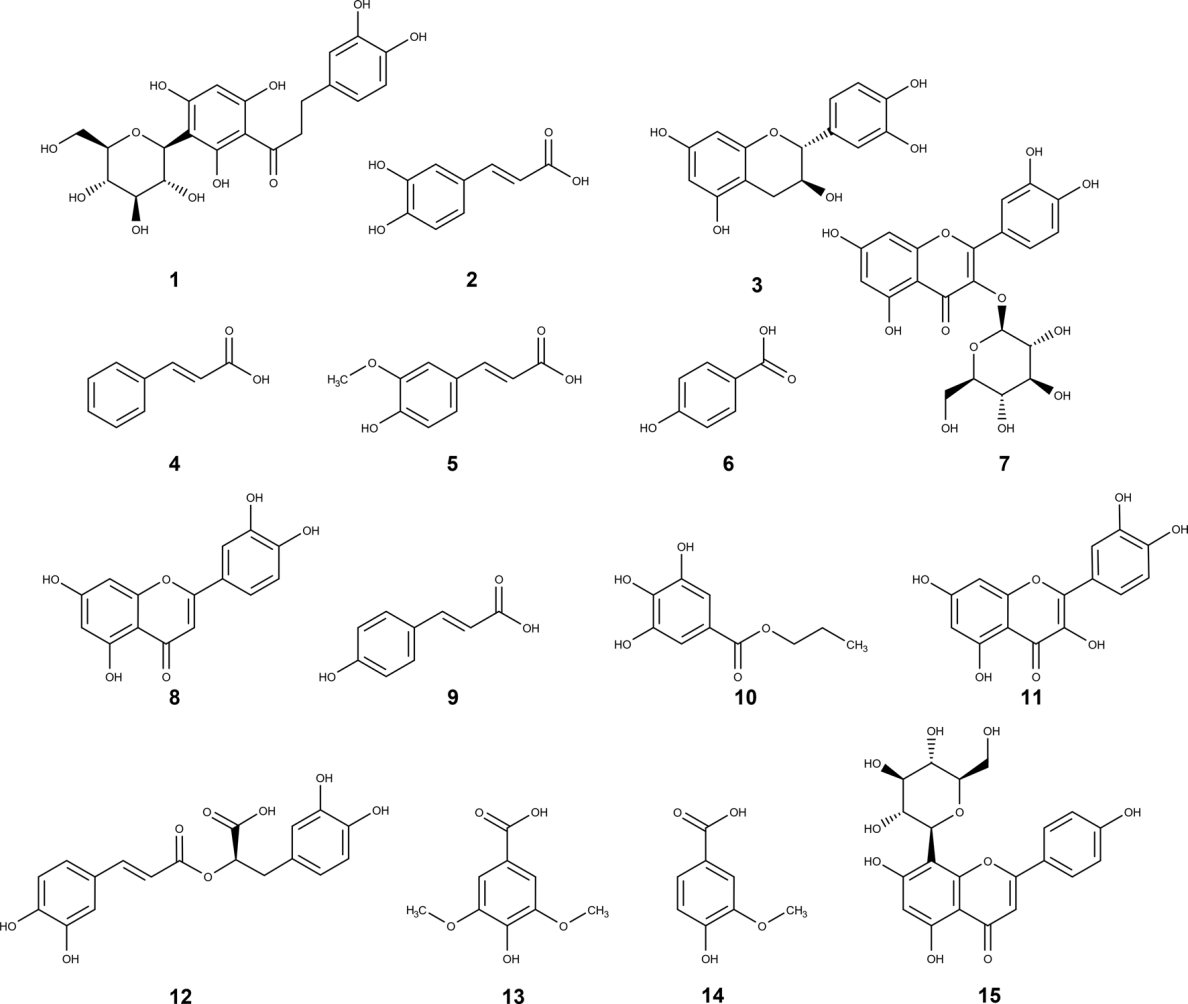
Compounds identified and quantified in the ethanolic extract of *Aspalathus linearis* using Ultra-Performance Liquid Chromatography—Quantitative Time of Flight analysis, namely aspalathin (**1**), caffeic acid (**2**), (+)-catechin (**3**), cinnamic acid (**4**), ferulic acid (**5**), 4-hydroxy benzoic acid (**6**), isoquercitrin (**7**), luteolin (**8**), p-coumaric acid (**9**), *n*-propyl gallate (**10**), quercetin (**11**), rosmarinic acid (**12**), syringic acid (**13**), vanillic acid (**14**) and vitexin (**15**).

Aspalathin (**1**) is the primary compound identified in the ethanolic extract of *Aspalathus linearis* (AL_EtOH_), which comprised of approximately 34% of the crude extract. The UPLC-QToF analysis indicated that only trace amounts of caffeic acid (**2**), ferulic acid (**5**), 4-hydroxybenzoic acid (**6**), p-coumaric acid (**9**) and *n*-propyl gallate (**10**) were present in AL_EtOH_. Small amounts of catechin (**3**), cinnamic acid (**4**), rosmarinic acid (**12**), syringic acid (**13**) and vanillic acid (**14**) were present (Fig. 1). The compounds present in higher amounts were isoquercitrin (**7**), luteolin (**8**), quercetin (**11**) and vitexin (**15**) (Table 1). The spectral data for Table 1 is available in section S1, Supporting Information. A link between the amount of the compounds present in AL_EtOH_, its fractions and their effect on the tyrosinase enzyme rate formation was determined.

**Table 1:** The quantification of major phyto-constituents present in the ethanolic extract of *Aspalathus linearis* and its fractions; using Ultra-Performance Liquid Chromatography—Quantitative Time of Flight.

Compounds	Quantification (µg_compound_/mg_sample injected_ ± SD_s_)						
	Crude extract	Hexane Fraction	DCM^b^ fraction	EtOAc^c^ fraction	EtOH^d^ fraction	AcOH^e^ fraction	Water fraction
Aspalathin (**1**)	338.96 ± 1.90	ND^a^	0.69 ± 0.82	704.08 ± 12.05	364.98 ± 5.85	95.14 ± 0.91	191.84 ± 2.68
Caffeic acid (**2**)	0.05 ± 0.003	0.02 ± 0.05	0.02 ± 0.05	0.02 ± 0.05	0.04 ± 0.004	0.04 ± 0.005	0.05 ± 0.004
Catechin (**3**)	0.79 ± 0.25	0.36 ± 0.18	0.32 ± 0.17	1.85 ± 0.44	1.84 ± 0.44	0.32 ± 0.17	0.46 ± 0.19
Cinnamic acid (**4**)	1.80 ± 0.82	1.00 ± 0.85	1.70 ± 0.82	2.21 ± 0.80	0.03 ± 0.88	2.15 ± 0.80	0.77 ± 0.85
Ferulic acid (**5**)	0.007 ± 0.01	0.022 ± 0.004	0.424 ± 0.05	0.411 ± 0.05	0.016 ± 0.01	0.031 ± 0.003	0.034 ± 0.002
4-Hydroxybenzoic acid (**6**)	3.07 ± 0.41	2.89 ± 0.38	0.57 ± 0.09	6.42 ± 0.83	4.02 ± 0.53	1.59 ± 0.22	4.60 ± 0.60
Isoquercitrin (**7**)	5.07 ± 1.10	8.92 ± 0.89	134.49 ± 6.02	ND^a^	8.61 ± 0.90	73.50 ± 2.67	ND^a^
Luteolin (**8**)	0.01 ± 0.02	0.02 ± 0.02	0.01 ± 0.02	0.09 ± 0.01	0.03 ± 0.02	0.06 ± 0.02	0.01 ± 0.02
p-Coumaric acid (**9**)	0.001 ± 0.003	ND^a^	0.145 ± 0.06	0.015 ± 0.003	ND^a^	0.020 ± 0.01	0.001 ± 0.003
*n*-Propyl gallate (**10**)	0.088 ± 0.004	0.088 ± 0.003	0.088 ± 0.003	0.094 ± 0.001	0.086 ± 0.004	0.085 ± 0.004	0.101 ± 0.001
Quercetin (**11**)	8.61 ± 0.43	7.37 ± 0.47	0.82 ± 0.67	14.68 ± 0.25	10.46 ± 0.37	4.57 ± 0.55	10.29 ± 0.38
Rosmarinic acid (**12**)	0.24 ± 0.09	0.08 ± 0.02	0.08 ± 0.02	0.11 ± 0.03	0.13 ± 0.04	0.09 ± 0.03	0.09 ± 0.03
Syringic acid (**13**)	0.12 ± 0.003	0.09 ± 0.004	0.09 ± 0.004	0.07 ± 0.01	0.09 ± 0.004	0.09 ± 0.004	0.07 ± 0.01
Vanillic acid (**14**)	0.12 ± 0.001	0.34 ± 0.001	0.24 ± 0.001	ND^a^	0.01 ± 0.001	ND^a^	0.13 ± 0.001
Vitexin (**15**)	5.61 ± 0.59	6.20 ± 0.69	0.56 ± 0.23	15.95 ± 2.27	9.64 ± 1.25	4.21 ± 0.36	9.48 ± 1.22

^a^*ND* not detected.

^b^Dichloromethane.

^c^Ethyl acetate.

^d^Ethanol.

^e^Acetic acid.

### Tyrosinase activity

The EtOAc fraction, the fraction with the highest levels of aspalathin (**1**), exhibited the highest enzyme stimulating effect resulting in a 0.67-fold increase in the enzymatic rate (Table 2). Similarly, the bioactivity of the other fractions had a positive correlation with the amount of aspalathin (**1**) present in the fractions, indicating that aspalathin (**1**) was the major compound responsible for the stimulatory effect of AL_EtOH_ on tyrosinase. Aspalathin (**1**) resulted in a significant increase (1.10-fold) in the enzymatic rate. Caffeic acid (**2**) and catechin (**3**), although only present in small amounts in AL_EtOH_, also exhibited a stimulatory effect on the enzyme rate with a 0.02 and 0.4-fold increase, respectively.

**Table 2:** Effect of the ethanolic extract, fractions and the phyto-constituents identified in *Aspalathus linearis* on tyrosinase rate.

Sample	Tyrosinase inhibition	Tyrosinase stimulation		Change in L-dopa kinetics	
	IC_50_ ± SD (µM)^a^	Fold increase^b^	EC_50_ ± SD (µg/mL; µM)^c^	*V*_max_^d^ (µM/min)	*K*_m_^e^ (µM)
**Substrate**					
L-dopa^f^	N/A^g^	N/A	N/A	22.82 ± 1.07	143.80 ± 25.43
**Extract**					
*A. linearis* EtOH extract	N/A	0.40	105.10 ± 2.03	61.86 ± 8.69	104.00 ± 45.97
**Fractions**					
Hex fraction	N/A	N/A	N/A	120.10 ± 4.41	371.40 ± 29.38
DCM fraction	N/A	N/A	N/A	11.68 ± 3.43	2732 ± 1027
EtOAc fraction	N/A	0.67	51.23 ± 1.33	111.80 ± 4.90	99.75 ± 11.67
EtOH fraction	N/A	0.50	140.60 ± 2.69	26.47 ± 0.76	149.60 ± 12.83
AcOH fraction	N/A	0.38	83.48 ± 2.35	18.37 ± 0.63	31.79 ± 4.23
H_2_O fraction	N/A	0.25	84.02 ± 1.59	181.30 ± 25.28	500.70 ± 138.3
**Compounds**					
Aspalathin (**1**)	N/A	1.10	119.70 ± 2.06 (264.75 ± 4.56 µM)	68.73 ± 3.47	1733 ± 660.60
Caffeic acid (**2**)	N/A	0.02	41.03 ± 3.54 (227.74 ± 19.65 µM)	132.00 ± 24.50	2810 ± 874.6
Catechin (**3**)	N/A	0.40	143.30 ± 2.74 (493.70 ± 9.44 µM)	382.40 ± 7.03	471.00 ± 26.43
Cinnamic acid (**4**)	943.58 ± 8.10	N/A	N/A	16.82 ± 0.37	269.90 ± 46.26
Ferulic acid (**5**)	> 1500	N/A	N/A	N/A	N/A
4-Hydroxybenzoic acid (**6**)	1868 ± 9.34	N/A	N/A	35.00 ± 1.84	267.20 ± 46.26
Isoquercitrin (**7**)	> 646.42	N/A	N/A^g^	N/A	N/A
Luteolin (**8**)	N/A	N/A	N/A	10.73 ± 1.01	232.10 ± 64.97
p-Coumaric acid (**9**)	152.58 ± 7.01	N/A	N/A	17.71 ± 0.66	254.00 ± 29.91
*n*-Propyl gallate (**10**)	534.87 ± 5.09	N/A	N/A	N/A	N/A
Quercetin (**11**)	> 992.60	N/A	N/A	20.56 ± 1.72	110.80 ± 33.67
Rosmarinic acid (**12**)	7.33 ± 3.33	N/A	N/A	N/A	N/A
Syringic acid (**13**)	> 1514	N/A	N/A	19.72 ± 1.01	401.10 ± 57.86
Vanillic acid (**14**)	> 1784	N/A	N/A	N/A	N/A
Vitexin (**15**)	> 693.83	N/A	N/A	N/A	N/A
Kojic acid^h^	14.79 ± 7.88	N/A	N/A	N/A	N/A

^a^Absolute IC_50_ value, the concentration exhibiting 50% inhibition of the enzyme’s activity.

^b^Determined as the enzyme rate of the sample divided by the enzyme rate of the vehicle control.

^c^Relative EC_50_ value, the concentration of the sample exhibiting 50% of its maximal response.

^d^*V*_max_ (maximum enzyme velocity).

^e^*K*_m_ (michaelis constant—concentration of the sample where the enzyme reaches half of its *V*_max_).

^f^Substrate.

^g^Not applicable/none active.

^h^Positive control.

The remaining compounds either inhibited the activity of the enzyme or had no noteworthy effect (Table 2). The Supporting Information and graphs for Table 2 are available in section S2, Supporting Information. Rosmarinic acid (**12**) exhibited noteworthy inhibition of the activity of the enzyme with an absolute IC_50_ value (the concentration resulting in 50% inhibition of the enzyme rate) of 7.33 ± 3.33 µM, which was comparable to the IC_50_ value of the positive control, kojic acid (14.79 ± 7.88 µM). Similarly, rosmarinic acid (**12**) has previously been reported for its antityrosinase activity with an IC_50_ value of 4.0 μM, exhibiting higher antityrosinase activity compared to that of the reported reference compounds; including kojic acid (14.28 ± 1.28 µM), hydroquinone (19.82 ± 1.05 µM, and resveratrol (27.67 ± 1.15 µM)^15,16^. Moderate inhibitory activity was reported for compounds **4**, **9** and **10**, which could indicate the lower effect of the extract on the enzyme activity compared to aspalathin (**1**). The type of inhibition for the compounds as mentioned earlier; were determined using enzyme kinetic studies indicating a competitive inhibition for compounds **4**, **7, 9, 12** and kojic acid, and non-competitive inhibition for *n*-propyl gallate (**10**). Well-known competitive and non-competitive inhibitors of tyrosinase are hydroquinone and resveratrol, respectively^17,18^. However, a recent study reported that resveratrol acts as a subversive substrate for tyrosinase, forming 4‐(3′,5′‐dihydroxy‐trans‐styrenyl)‐1,2‐benzoquinone^19^. Resveratrol and caffeic acid have similar structures, therefore, the stimulation observed for caffeic acid could be due to the compound acting as a subversive substrate.

Enzyme kinetics, in the absence of the substrate, were used to determine whether the tyrosinase rate increase exhibited by compounds **1**, **2** and **3**; was due to their subversive substrate activity on tyrosinase (Table 3). Compounds **1**, **2** and **3** resulted in significantly high V_max_ values of 68.7 ± 3.5, 120.9 ± 15.5 and 382.4 ± 7.0 µM/min, respectively, as compared to the V_max_ value of 22.82 ± 1.07 µM/min for the substrate L-dopa. However, the K_m_ values of 1733 ± 660.6, 2428 ± 553.6 and 471.0 ± 26.4 µM for compounds **1**, **2** and **3**, respectively, were much higher than that of the native substrate (143.8 ± 25.4 µM), indicating that larger quantities are necessary for the enzyme to exhibit the same rate. Further analysis on the catalytic conversion and substrate catalytic efficiency revealed that catechin (**3**), exhibited the highest substrate catalytic conversion (4 times higher efficiency compared to the native substrate, L-dopa). Although, aspalathin (**1**) and caffeic acid (**2**), both had subversive substrate activity, their efficiency were approximately 3–4 times lower than the native substate. This is the first report and analysis on the substrate properties of compounds **1**–**3** on tyrosinase.

**Table 3:** Substrate properties of compounds from *Aspalathus linearis*, on tyrosinase.

Compound	*K*_m_^a^ (µM)	*V*_max_^b^ (mOD/min)	*k*_cat_^c^ (s^-1^)	*k*_cat_/*K*_m_^d^ (× 10^3^)
L-dopa^e^	143.8 (± 25.4)	22.8 (± 1.1)	13.7	95
Aspalathin (**1**)	1733 (± 660.6)	68.7 (± 3.5)	41.3	24
Caffeic acid (**2**)	2428 (± 553.6)	120.9 (± 15.5)	72.6	30
Catechin (**3**)	471 (± 26.4)	382.4 (± 7.0)	229.7	488

^a^Michaelis constant.

^b^Maximum velocity.

^c^Catalytic constant.

^d^Catalytic efficiency.

^e^Native substrate.

**Figure 2:**
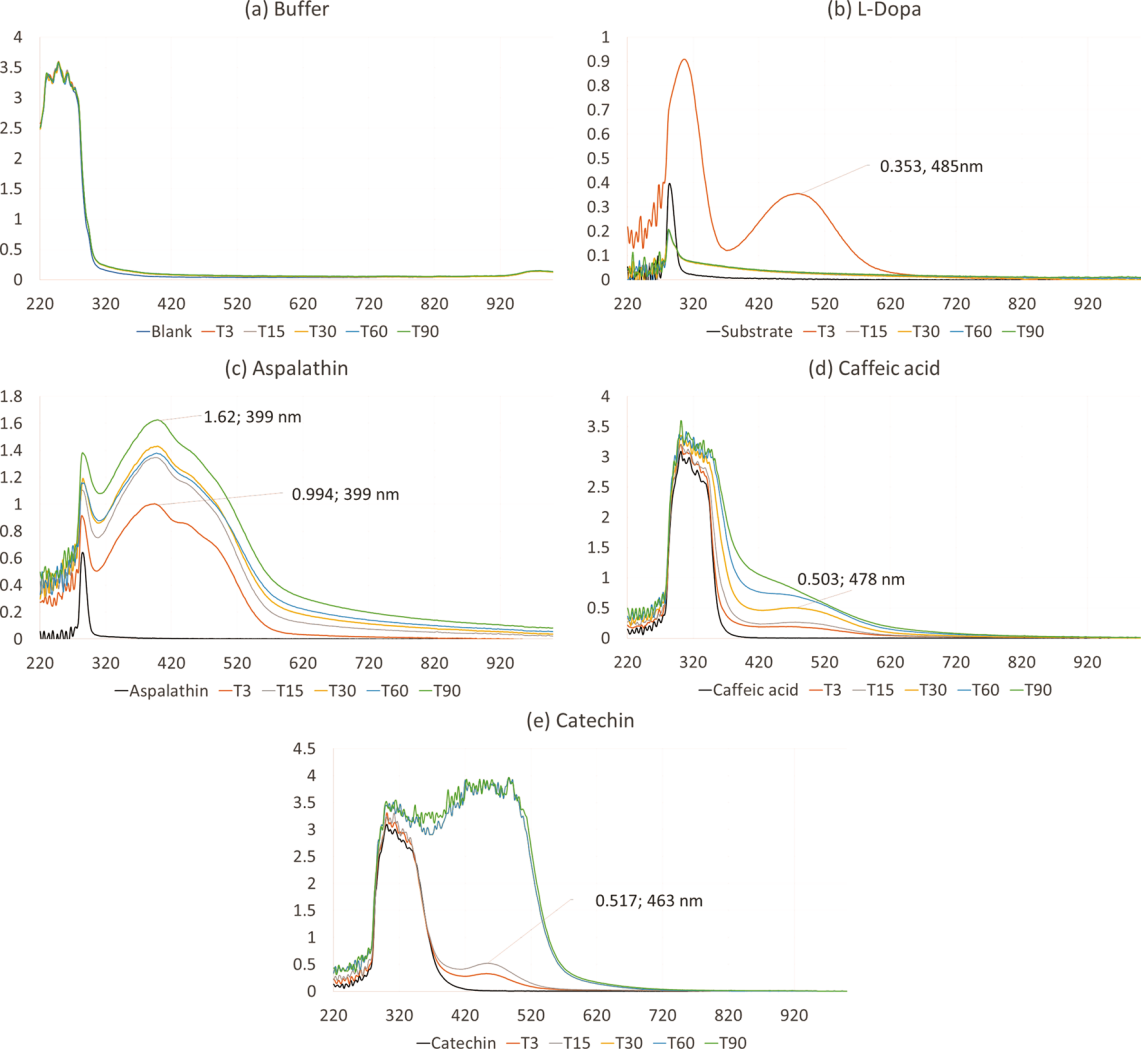
Ultraviolet spectral scan representing the interaction of the (**a**) buffer as control (**b**) substrate (L-dopa), and subversive substrates (**b**) aspalathin (**1**), (**d**) caffeic acid (**2**) and (**e**) catechin (**3**) with the enzyme over 90 min (T3–T90). The black line indicates the samples in the absence of the enzyme. The substrate, L-dopa, was oxidised to L-dopa-chrome, indicated by the peak at 485 nm at 3 min of assay run-time. The formation of L-dopa-chrome was limited to the amount of substrate available, leading to the flattening of the curve.

The UV-spectral scan of the substrate, L-dopa, after 3 min of assay run-time, indicated a product with a λ_max_ peak at 485 nm, possibly representing L-dopa-quinone, while aspalathin (**1**) exhibited a peak at 399 nm, caffeic acid a peak at 478 nm, and catechin (**3**) exhibited a peak at 463 nm, indicating that different products for the respective subversive substrates were formed compared to that of the native substrate (Fig. 2). Tyrosinase has been reported for oxidising the hydroxy or dihydroxy groups to form cyclic dione structures, namely quinones^20,21^. The data potentially suggest that tyrosinase catalyses the oxidation of the dihydroxybenzyl moiety of aspalathin (**1**) to form an *ortho*-benzoquinonyl group. These compounds, produced by the oxidation of aspalathin (**1**), have been reported for their browning effect in fermented rooibos^22^. Similarly, oxidised catechin (**3**) isomers have been reported for their colouration with absorbances at 380, 390 and 420 nm^23^. The dihydroxybenzyl moiety of caffeic acid interacted with the enzyme to form a product observed at 478 nm.

It has been reported that the presence of Cu^2+^ ions facilitates the kinetics of the cyclisation of dopa-chrome, which is a vital step in melanogenesis. Therefore, it was hypothesised that the copper in AL_EtOH_, or rather the oxidised form Cu^2+^, is available for the conversion of dopa-chrome, which indirectly stimulated the tyrosinase enzyme^24^. It was determined that copper was present in the ethanolic extracts (AL_EtOH_) at 0.060 ± 0.034 mg/kg (S3, Supporting Information). Therefore, apart from the increased rate from compounds **1** and **3** acting as subversive substrates, higher levels of copper ions within the extract could also facilitate the higher enzymatic rate observed.

### Computer modelling

**Figure 3:**
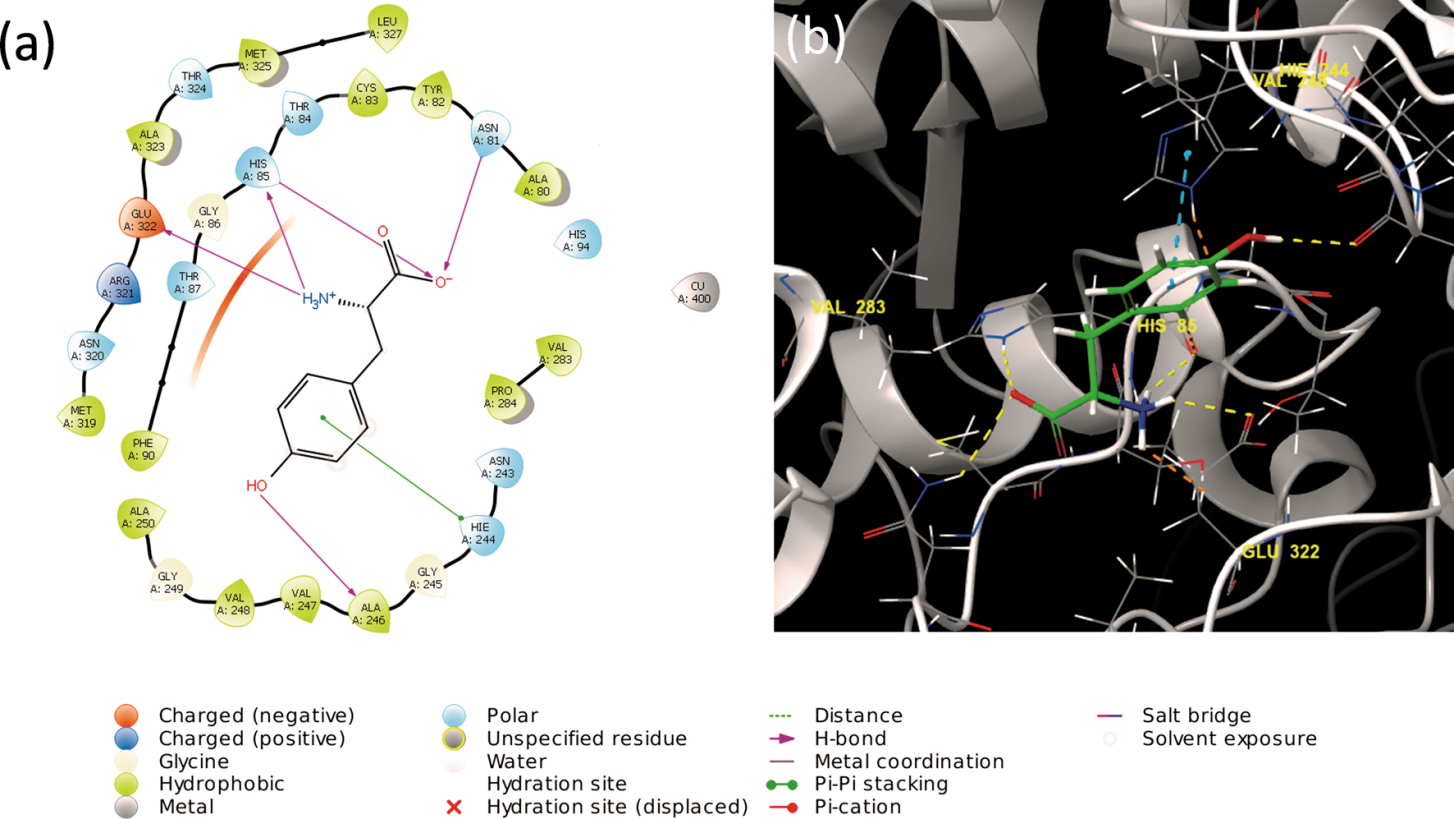
(**a**) Two- and (**b**) three-dimensional ligand interaction diagrams of L-tyrosine docked within the tyrosinase structure (PBD: 2Y9X). The blue dashed line in image (**b**) represents pi-pi stacking, while the yellow and orange dashed lines represents different hydrogen bonds between the compounds and the active site residues of the enzyme (https://www.schrodinger.com/maestro; version 12.1.013, release 2019–3).

**Figure 4:**
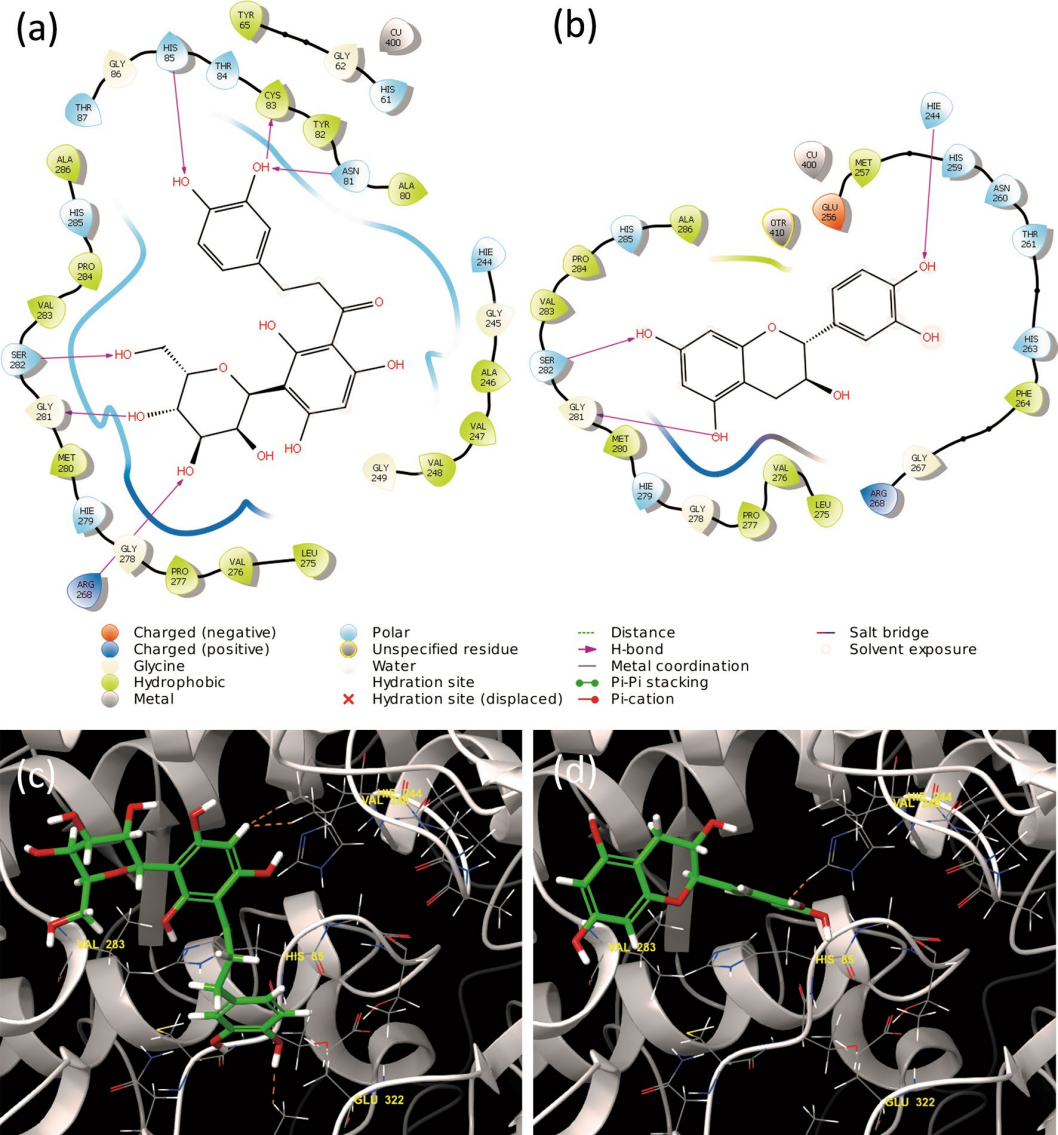
Two- and three-dimensional ligand interaction diagrams of (**a**, **c**) aspalathin (**1**) and (**b**, **d**) catechin (**3**) using the tyrosinase structure (PBD: 2Y9X). The primary bonds formed are hydrogen bonds between aspalathin (**1**) and asparagine 81, cysteine 83, histidine 85, serine 292, glycine 291 and arginine 268, and catechin (**3**) and histidine 244, serine 282 and glycine 281. Both aspalathin (**1**) and catechin (**3**) stimulated the tyrosinase enzyme rate in vitro. Hydrogen bonds are represented by orange dashed lines in images (**c**) and (**d**) (https://www.schrodinger.com/maestro; version 12.1.013, release 2019-3).

**Figure 5:**
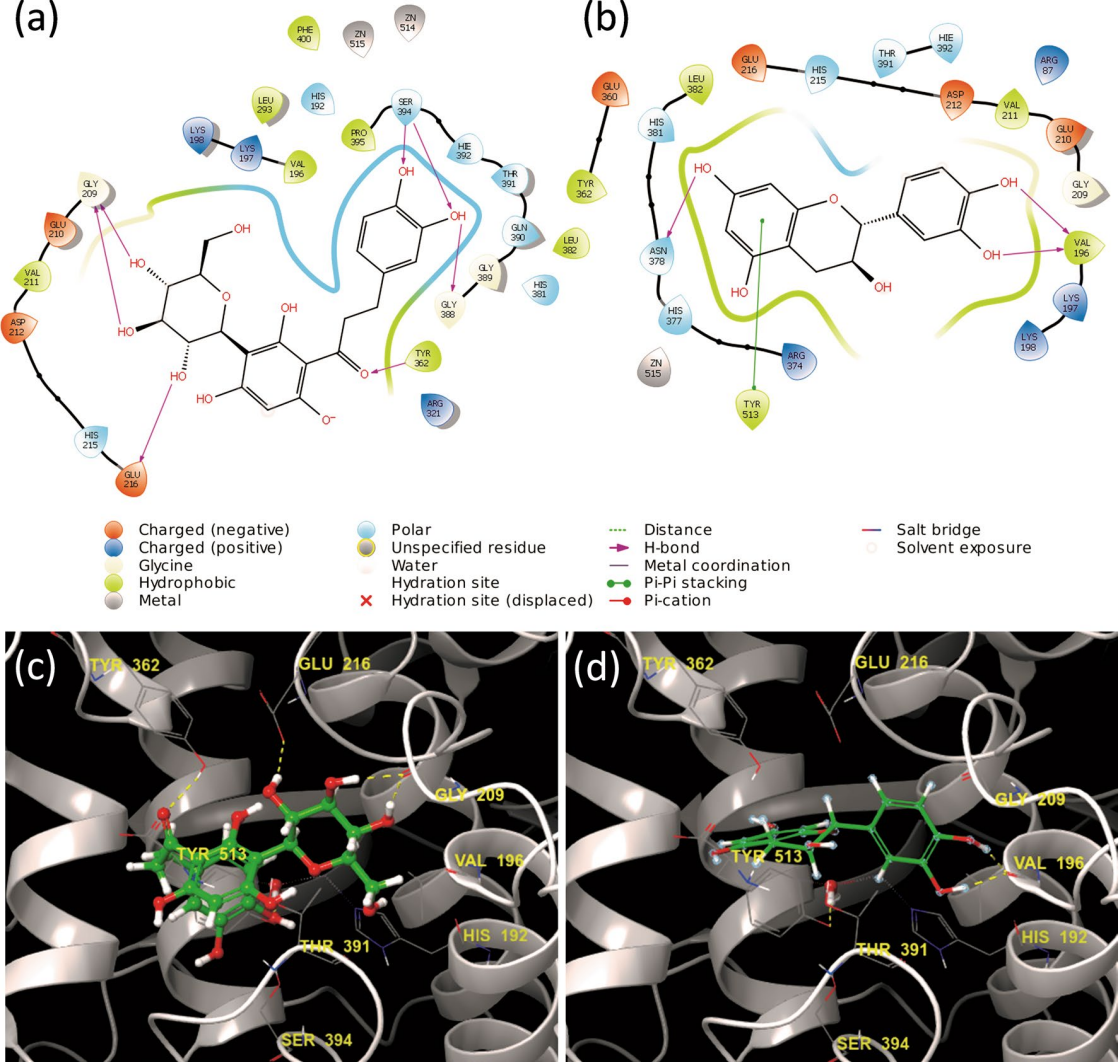
Two- and three-dimensional ligand interaction diagrams of (**a**, c) aspalathin (**1**) and (**b**, **d**) catechin (**3**) using the tyrosinase-related protein 1 structure (PBD: 5M8P). The primary bonds formed are hydrogen bonds between aspalathin (**1**) and tyrosine 362, glycine 209 and glutamine 216, and catechin (**3**) and valine 196, threonine 391, tyrosine 362 and asparagine 378, and pi-pi stacking of the benzyl ring with histidine 215. Hydrogen bonds are represented by yellow dashed lines in images (**c**) and (**d**) (https://www.schrodinger.com/maestro*;* version 12.1*.*013, release 2019-3).

The ligand–protein interaction was predicted using molecular docking. The corresponding Glide XP Score for each compound was used to determine the most favourable binding pose and the potential affinity of the ligand to the active site. The ligand–protein interaction of compounds **1** to **15** is available in section S4, Supporting Information. The docking score represents the energy interactions between the ligand and the active site, combining different parameters to identify potential binding. These parameters include, van der Waals interactions, hydrogen bonding, coulomb energy, lipophilic and metal contact binding to name a few. The binding energy is dependent on the type of bonds present between the ligand and the active sites, including hydrogen bonds, pi-pi stacking, cation-pi bonds and salt bridges (Figs. 3, 4, 5). A lower value indicated greater stability of the binding affinity between the ligand and protein; therefore, the lower the value, the better the probable affinity. All the compounds bound to both the tyrosinase (PBD: 2Y9X) and tyrosinase related protein 1 (PBD: 5M8P) structures; however, only the docking scores of compounds **1** and **7** indicated a strong potential binding affinity with 2Y9X, and compounds **1**, **2**, **3**, **7**, **8**, **12**, with 5M8P.

Tyrosinase related protein 1 catalyses the conversion of 5,6-dihydroxyindole-2-carboxylic acid, which is formed from dopa-chrome via dopa-chrome tautomerase, to indole-5,6-quinone carboxylic acid that ultimately forms eumelanin. Tyrosinase related protein 1 structure (PBD: 5M8P) was included in the docking study to determine whether the compounds have similar affinity to tyrosinase (PBD: 2Y9X).

Compounds **1**, **2**, **3**, **7**, **8**, **11**, **12** and **15** exhibited greater stability for both the 2Y9X and 5M8P structures than kojic acid, the positive control, the reference compound, and L-tyrosine (Table 4, Fig. 3). No clear correlation could be drawn from the average hydrogen bond length or number of hydrogen bonds observed for the compounds and their stability for both the 2Y9X and 5M8P structures (S4, Supporting Information, Table S3, S4).

**Table 4:** Docking scores of the compounds using the 2Y9X tyrosinase structures and 5M8P tyrosinase related protein 1 and the mushroom tyrosinase activity (%) of the compounds at a concentration of 200 µg/mL.

Compounds	Glide XP docking scores		Tyrosinase activity at 200 µg/mL (fold increase)^b^
	2Y9X	5M8P	
Aspalathin (**1**)	− 8.88	− 10.48	1.11
Caffeic acid (**2**)	− 3.63	− 6.48	− 0.01
Catechin (**3**)	− 3.62	− 6.13	0.41
Cinnamic acid (**4**)	− 2.00	− 3.25	− 0.51
Ferulic acid (**5**)	− 2.99	− 4.80	− 0.35
4-Hydroxybenzoic acid (**6**)	− 2.71	− 5.31	− 0.11
Isoquercitrin (**7**)	− 7.19	− 9.27	− 0.20
Luteolin (**8**)	− 4.36	− 6.36	− 0.15
*p*-Coumaric acid (**9**)	− 2.51	− 5.25	− 0.99
*n*-Propyl gallate (**10**)	− 5.11	− 5.01	− 0.70
Quercetin (**11**)	− 4.40	− 5.88	− 0.61
Rosmarinic acid (**12**)	− 4.58	− 8.82	− 0.83
Syringic acid (**13**)	− 3.99	− 4.44	− 0.24
Vanillic acid (**14**)	− 4.24	− 4.08	− 0.22
Vitexin (**15**)	− 5.21	− 7.18	− 0.32
Kojic acid^a^	− 3.12	− 4.69	− 0.98
L-tyrosine	− 3.82	N/A	N/A
Reference compound	− 3.21	− 5.63	N/A

^a^Positive control for the tyrosinase assay

^b^Determined as the enzyme rate of the sample divided by the enzyme rate of the vehicle control; the fold increase values of the samples were recorded at 200 µg/mL. The enzyme rate was kinetically determined over a period of 30 min at 37 °C.

Aspalathin (**1**) exhibited the greatest stability and potential affinity with the lowest docking score in both 2Y9X and 5M8P, which correlated positively with the observed stimulatory effect on tyrosinase. Comparably, isoquercitrin that has a similar structure to aspalathin exhibited subsequently best stability. The strong binding affinity of the compound could be ascribed to the presence of hydroxy, carbonyl and ethereal groups forming hydrogen bonds. Several hydrogen bonds were formed between the hydroxy groups present in aspalathin (**1**) and isoquercitrin (**7**), with the histidine 85, serine 292, glycine 291 and arginine 268 amino acids present in the active site of 2Y9X. Vitexin, which exhibited the third best stability formed a hydrogen bond between a hydroxy group and glutamine 322, and the ketone group and valine 283. A pi-pi stacking interaction between the phenol group and phenylalanine 264 was observed. However, pi-pi stacking interaction did not have a significant effect on the stability of the compounds. Based on the different hydrogen bonds formed for the 15 compounds the residues primarily responsible for successful docking with 2Y9X were asparagine 81, cysteine 83, histidine 85, serine 292, glycine 291 and arginine 268. The corresponding residues identified from the reference ligand, L-tyrosine, were histidine 85 and asparagine 81, suggesting that these residues are important for the catalysis. The docking pose of aspalathin (**1**) was compared to L-tyrosine and showed similar binding and alignment of the core structure to the binding site (S4, Supporting Information, Figure S7).

On the other hand, the different hydrogen bonds formed between the 15 compounds and the residues primarily responsible for successful docking with 5M8P were asparagine 81, cysteine 83, glycine 281 and alanine 246. Aspalathin (**1**) formed similar hydrogen bond interactions with 5M8P as observed with 2Y9X. The primary hydrogen bonds formed in 5M8P between aspalathin (**1**) and asparagine 81, cysteine 83, histidine 85, serine 292 and glycine 291, were similar to 2Y9X, except no hydrogen bond formed between aspalathin (**1**) and arginine 268. The aforementioned similarity indicated that the active site of tyrosinase and tyrosinase related protein 1 are similar and compounds that successfully docked in the one protein would successfully dock in the other.

The relationship between the docking scores and the tyrosinase activity exhibited by the compounds were analysed using the Pearson correlation, assuming a Gaussian distribution. The full data set is available in section S5, Supporting Information. The correlation coefficient between the two docking scores was 0.7618 indicating that the two variables tend to increase together. The correlation was found to be significant with a *p* value lower than 0.001. Therefore, it can be concluded that the compounds might have a similar effect on the tyrosinase related protein 1 as with tyrosinase. The correlation coefficient was negative for the correlation between the tyrosinase activity and the docking scores for the tyrosinase structure with an r-value of -0.5696 (*p* value < 0.05). The correlation indicated that as the one variable increased the other decreased. Therefore, the lower the Glide XP Score, the higher the fold-increase in enzyme activity.

### Quantitative structure–activity relationship model

**Figure 6:**
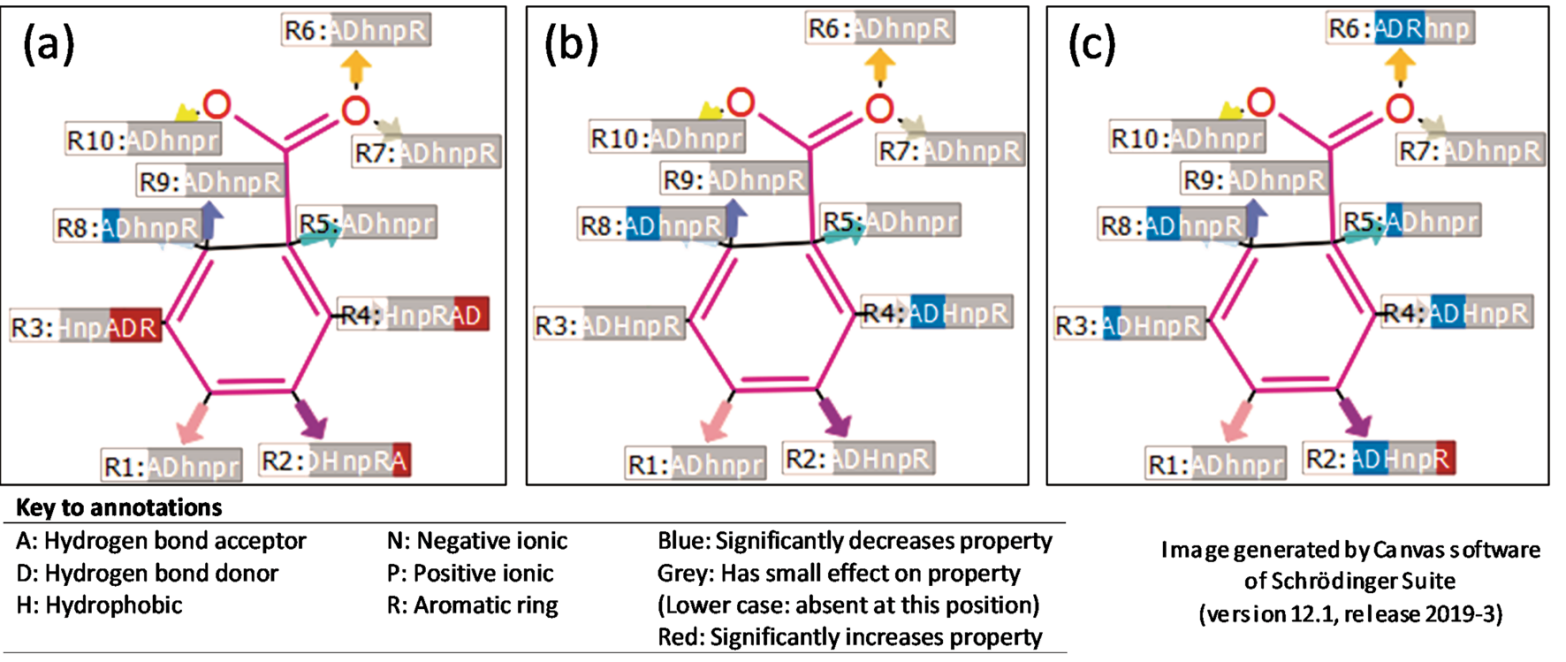
Pharma rational quantitative structure–activity relationship models indicating the R-groups most likely responsible for the (**a**) tyrosinase activity, (**b**) tyrosinase structure (PBD: 2Y9X) docking score properties and (**c**) tyrosinase related protein 1 structure (PBD: 5M8P) docking score properties. According to the six pharmacophore elements analysed, the substituent groups at the R2 position (hydrogen bond acceptor), R3 (hydrogen bond acceptor, hydrogen bond donor and aromatic ring) and R4 (hydrogen bond acceptor and hydrogen bond donor) had a significant increase on the tyrosinase activity. The substituent groups at the R8 position (hydrogen bond acceptor) had a significant decreasing effect on the activity. The substituent groups at the R4 position (hydrogen bond acceptor and hydrogen bond donor) and R8 position (hydrogen bond acceptor and hydrogen bond donor) had a significant decreasing effect on the tyrosinase structure docking score properties. The substituent groups at the R2 position (hydrogen bond acceptor and hydrogen bond donors), R3 (hydrogen bond acceptor), R4 (hydrogen bond acceptor and hydrogen bond donors), R5 (hydrogen bond acceptor), R6 (hydrogen bond acceptor, hydrogen bond donors and aromatic ring), and R8 (hydrogen bond acceptor and hydrogen bond donor) had a significant decreasing effect on the tyrosinase-related protein 1 structure docking score properties. In contrast, the substituent groups at R2 (aromatic ring) had a significant increasing effect (https://www.schrodinger.com/canvas; version 4.1.013, release 2019-3).

R-group analysis was done on the tyrosinase activity and docking score properties of the 15 compounds. The data obtained with the QSAR model is available in S6, Supporting Information. A core scaffold was determined, and 10 R-groups were identified. According to the six pharmacophore elements analysed, the substituent groups at R2, R3, R4 and R8 had a significant effect on tyrosinase activity (Fig. 6). The Pharma RQSAR was done on the tyrosinase activity recorded at 200 µg/mL of the compound (Table 4). The elements resulting in a significant increase in the tyrosinase property at the different positions were as follows, R2: hydrogen bond acceptor, R3: hydrogen bond acceptor, hydrogen bond donor and aromatic ring, and R4: hydrogen bond acceptor and hydrogen bond donor. The property, at the R8 position, resulting in a significant decrease in tyrosinase activity was identified as a hydrogen bond acceptor. Aspalathin (**1**) has several hydrogen bond donors in the form of trihydroxybenzyl and dihydroxybenzyl groups, which could be linked to its bioactivity. Catechin (**3**), on the other hand, has a benzopyran group at position R3 with three hydrogen bond donors (–OH), which is possibly responsible for its bioactivity. These hydrogen bonds present in compounds **1** and **3** is most probably the groups oxidised by tyrosinase to form their respective products. On the other hand, rosmarinic acid (**12**), a tyrosinase inhibitor preventing product formation, contains several hydrogen bond acceptors in the form of hydroxy, carbonyl and ethereal groups at its R8 position, resulting in a significant reduction in the enzymatic rate. Although the R-group analysis revealed no hydrogen bond acceptors for kojic acid at the R8 position, its inhibitory activity probably lies in that it is a small compound that can easily fit into the active site of tyrosinase together with its polar interaction with Cu^2+^ ions^25^.

The elements resulting in a significant decrease in docking score property for the tyrosinase structure at the different positions were as follows, R4: hydrogen bond acceptor and hydrogen bond donors, and R8: hydrogen bond acceptor and hydrogen bond donor. The elements resulting in a significant decrease in docking scores for the tyrosinase related protein 1 structure at the different positions were as follows, R2: hydrogen bond acceptor and hydrogen bond donors, R3: hydrogen bond acceptor, R4: hydrogen bond acceptor and hydrogen bond donors, R5: hydrogen bond acceptor, R6: hydrogen bond acceptor, hydrogen bond donors and aromatic ring, and R8: hydrogen bond acceptor and hydrogen bond donor. Aspalathin (**1**) exhibited the best docking score in both 2Y9X and 5M8P, followed by isoquercitrin (**7**).

Apart from the trihydroxybenzyl and dihydroxybenzyl groups of aspalathin (**1**) at position R4, there is a hydroxy group at the R5 position further increasing the docking score property. Similarly, isoquercitrin (**7**) contains a dihydroxybenzyl group as well as a benzopyran group with several hydrogen bond acceptors and hydrogen-bond donors at the R4 position, together with a hydroxy group at the R5 position. Rosmarinic acid (**12**), the compound with the third-lowest docking score in the 5M8P, contains several hydrogen bond acceptors, including an ester, carboxylate and dihydroxybenzyl group at its R8 position.

The activity cliffs did not reveal any significant deviation between the different compounds for the tyrosinase property and docking score properties that could indicate small structural differences potentially responsible for the compound’s bioactivity.

The enzyme reaction is not the only aspect to consider when investigating melanin production. The effect of the compounds on melanogenesis within human melanocytes, were analysed using the Masson Fontana assay. This assay makes use of the oxidation of silver nitrate by melanin to determine the effect on melanogenesis. Melanogenesis leads to the production of black-brown pigments (eumelanin) and red-yellow pigments (pheomelanin)^26^. It has been reported that the different skin types are not dependent on the ratio of eumelanin to pheomelanin, but rather the number of melanin pigments present in the epidermis^27^.

### Antiproliferation and Masson Fontana assays

**Figure 7:**
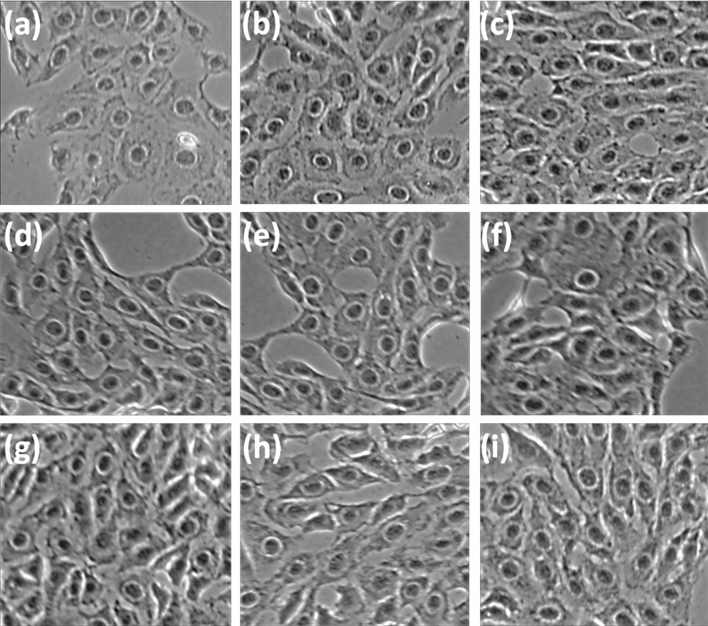
Light microscopic micrographs at 20X magnification of human melanocytes (UCT-Mel-1) stained with silver nitrate during the Masson Fontana assay. The dark stained areas indicated melanin present within the cells. The samples were compared with the (**a**) untreated cell control. (**b**) Alpha melanocyte-stimulating hormone (positive control) resulted in an increase of 9.73%, (**c**) ethanolic extract of *Aspalathus linearis* (AL_EtOH_) with an increase of 5.18%, (**d**) acetic acid fraction of the AL_EtOH_ with an increase of 4.87%, (**e**) ethyl acetate fraction of AL_EtOH_ with an increase of 7.04%, (**f**) ethanol fraction of AL_EtOH_ with an increase of 4.78%, (**g**) water fraction of AL_EtOH_ with an increase of 5.67%, and (**h**) aspalathin (**1**) and (**i**) catechin (**3**) that resulted in an increase of approximately 4.5%.

The extract of *A. linearis*, its fractions, and the compounds aspalathin (**1**) and catechin (**3**) exhibited no observable effect on cellular proliferation with 50% cell viability concentrations higher than 200 µg/mL, indicating low to no toxicity. The samples were tested at 100 µg/mL in the Masson Fontana assay (Table 5). The full data sets are available in section S7 and S8, of the Supporting Information. All samples exhibited a statistically significant (*p* value < 0.001) melanin stimulatory effect compared to the untreated cells (Fig. 7). The EtOAc fraction had the highest stimulatory effect with a 7.04% increase in melanin produced, followed by the AcOH fraction (5.18%), EtOH fraction (4.78%) and H_2_O fraction (3.90%). This cellular-based result correlates with the tyrosinase activity observed previously, supporting the probable mechanism. This trend suggests that the increase in melanogenesis is due to the increased tyrosinase activity. Aspalathin and catechin (**3**) were tested at 265 µM (119.82 µg/mL and 76.92 µg/mL, respectively), and both exhibited a significant increase of approximately 4.5% in the melanin produced.

**Table 5:** Antiproliferative analysis against human melanocytes and the percentage melanin produced by the EtOH extract of *Aspalathus linearis*, its fractions and the compounds, exhibiting an increase in the tyrosinase enzyme rate.

Samples	Antiproliferative IC_50_^c^ (µg/mL) ± standard deviation	Masson Fontana % increase in melanin produced (100 µg/mL^d^)
*A. linearis* EtOH extract	345.50 ± 2.47	5.18%
AcOH fraction	208.50 ± 1.08	4.87%
EtOAc fraction	231.70 ± 1.09	7.04%
EtOH fraction	230.70 ± 1.09	4.78%
H_2_O fraction	359.60 ± 1.15	5.67%
Aspalathin (**1**)	217.75 ± 1.23 (481.60 µM)	3.77%
Catechin (**3**)	166.32 ± 1.14 (573 µM)	5.72%
αMSH^a^	> 100 (60 µM)	9.73%^e^
Actinomycin D^b^	0.039 ± 0.002	N/A

^a^Positive control for the Masson Fontana assay.

^b^Positive control for the antiproliferative assay.

^c^Concentration where 50% of the cells are viable.

^d^Positive control tested at 30 µg/mL (18 µM).

## Conclusion

This study indicates that multiple factors contribute to the change in melanogenesis, due to *Aspalathus linearis*. Firstly, the increase in the rate of tyrosinase exhibited by the extracts could potentially be due to the increased number of copper ions available, leading to an increase formation of 5,6-dihydroxyindole-2-carboxylic acid and ultimately eumelanin. Secondly; the increase in the rate of tyrosinase exhibited by aspalathin (**1**) and catechin (**3**) was most probably due to their subversive substrates activity and not because of the increased conversion rate of L-dopa to L-dopaquinone. These compounds reacted with tyrosinase to form different products that have been reported for their browning effect. Therefore, it is possible that these compounds were present in the cells and oxidised by the silver nitrate in the Masson Fontana assay, forming their respective oxidative forms that could be responsible for the colour change observed. It is still unknown whether this phenomenon could potentially lead to skin browning and thereby act as a possible treatment for hypopigmentary disorders. A clinical investigation, in the form of a topical application, is required to investigate the effect of the extract and phytochemicals on melanogenesis in a in vivo setting.

## Experimental section

### Plant material, extraction and fractionation

Dried leaves and twigs of green *A. linearis* were donated by Rooibos Ltd., (batch number 588) Clanwilliam (S 32° 11.131′ EO 18° 53.291′). A herbarium voucher specimen (PRU: 122176) was deposited at the H.G.W.J. Schweickerdt Herbarium, University of Pretoria, South Africa. The coarsely ground (0.4 mm) plant material (13.85 kg) was extracted with 34 L of EtOH. The ethanolic extract of *A. linearis* (AL_EtOH_) was filtered with a Buchner funnel (Whatman No. 3 filter paper), concentrated using a rotary evaporator at 40 °C and freeze-dried to a fine powder. The percentage yield of the freeze-dried extract was 9.03% of the dried plant material. One hundred grams of the ethanolic extract (was dissolved in *n*-hexane (8 L), and sequentially and exhaustively partitioned in different solvents, including *n*-hexane (8 L), DCM (3 L), EtOAc (3 L), EtOH (5 L), AcOH (0.5 L) and H_2_O (5 L). Fifteen commercially available compounds (purchased from Sigma Aldrich, St. Louis, Missouri, United States) were quantified in the extract and the fractions using UPLC-QTOF analysis.

### Quantification of pure compounds using ultra-performance liquid chromatography: quantitative time of flight

Fifteen compounds previously isolated from *A. linearis* were quantified in AL_EtOH_ and its major fractions using a Waters Synapt G2 QTOF system and following the method described by Wooding et al.^28^. The compounds present in AL_EtOH_ and its major fractions were quantified by plotting a standard curve of each pure compound at five concentrations (250 µg/mL, 25 µg/mL, 2.5 µg/mL, 0.25 µg/mL, 0.025 µg/mL)^28^.

### Tyrosinase assay

The diphenolase tyrosinase assay was based on the method described by van Staden et al.^29^, with slight modifications. The enzymatic rate of mushroom tyrosinase (Sigma-Aldrich Co. LLC, St. Louis, MO, USA) was evaluated spectrophotometrically using L-dopa as a substrate. The formation of L-dopa-quinone was kinetically measured every 30 s for 30 min at 37 °C and detected at 492 nm, using a BIO-TEK Power-Wave XS multi-well plate reader (A.D.P., Weltevreden Park, RSA). A 96-well plate was prepared containing phosphate buffer (pH 6.5), 5% DMSO, substrate (637.50 µM), enzyme (33.30 units/mL) and the sample ranging from 3.13 to 200 µg/mL. DMSO (5%) was used as the vehicle control and kept constant at all tested concentrations, while kojic acid served as the positive control (0.78–50 µg/mL)^29^.

### Computer modelling

The ligands were docked to the target proteins using the Glide module within the Schrödinger Maestro program (version 12.1, release 2019-3). The ligands were retrieved in their mol format from ChemSpider (S4, Supporting Information, Table S2) and were prepared using LigPrep by selecting the OPLS3e force field. Different possible ionisation states were generated using Epik at a pH of 7 ± 2^30^. Tautomers and stereoisomers were generated at a maximum of 32 per ligand while retaining the specified chiralities. The enzyme structures were obtained from the protein data bank (PDB id: 2Y9X and 5M8P). The active sites were identified using the tropolone ligand, a known inhibitor of tyrosinase, and tyrosine that formed part of the 2Y9X and 5M8P PDB files. The missing residues for the 2Y9X and 5M8P proteins were 56 and 0, respectively. The missing heteroatoms for some of the residues for the 2Y9X and 5M8P proteins were 0 and 24, respectively. The Protein Preparation wizard was used for the enzyme pre-processing steps, which included the removal of water, assignment of bond orders, the addition of hydrogens, the creation of disulfide bonds and the generation of Het states using Epik (pH 7 ± 2). The hydrogen bonds were optimized using PROPKA with the pH set at 7 ± 2. Finally the restrained minimisation was done using OPLS3e^31,32^. A grid, similar in size to the crystal structure ligands, was created to facilitate the ligand docking in the active site of the enzyme using the receptor grid generation module. The grid size was set to 10 × 10 × 10 Å, selecting the reference ligand as the centroid, while setting the van der Waals scaling factor to one with a partial charge cut-off of 0.25. For the docking experiment, the extra precision (XP) mode of Glide, with the ligand sampling set to flexible was used. The van der Waals scaling was set to 1.0, and the partial charge cut-offs was set to 0.25. The root mean square deviation (RMSD) from the input geometries of the reference ligands were calculated and used for the docking protocol validation. A RMSD of lower than 1.5 Å, was considered to be a successful protocol.

### Quantitative structure–activity relationship model

R-group analysis, of the 15 compounds, previously identified in *A. linearis,* was performed using the Canvas software of Schrödinger Suite (version 12.1, release 20193). The core of the 15 compounds were obtained from a Canvas MCS run, which determined the maximum common substructure. The option of ‘evaluating all the equivalent atoms and all equivalent bonds’ was selected as the atom typing scheme. The tyrosinase activity and docking score properties of the compounds were analysed using the Pharma RQSAR model. This model annotates each R-group according to ther pharmacophore features namely, hydrogen-bond acceptor (A), hydrogen-bond donor (D), hydrophobic (H), negatively charged (N), positively charged (P), and aromatic (R). The R-groups that significantly decreases and increases the property was indicated. During the Pharma RQSAR model, the error was set as 0.30, and the importance was set as 2.79, 0.10 and 0.12 for the tyrosinase activity. tyrosinase (PBD: 2Y9X) and tyrosinase related protein 1 (PBD: 5M8P) docking score properties, respectively. The structure–activity relations were further investigated using “activity cliffs”, to identify whether small structural changes are resulting in significant deviations in the compound’s activity. The analysis was done with the fingerprint settings set to atom-type differences.

### Antiproliferative assay

The antiproliferative activity of AL_EtOH_, the fractions, and compounds was determined on human melanoma cells (UCT-Mel1). The cells were maintained in Dulbecco's Modified Eagle's Medium (DMEM), supplemented with 10% fetal bovine serum (FBS) and 1% antibiotics (Penicillin–Streptomycin). Cells were seeded in 96-well plates (100,000 cells/mL) and incubated overnight at 37 °C in 5% CO_2_ to allow for attachment. Following incubation, the cells were treated with varying concentrations of the samples (1.56–200 µg/mL) and the positive control (actinomycin D, 0.0039–0.5 µg/mL) for 72 h at 37 °C in 5% CO_2_. An untreated cell control and solvent control (DMSO 0.5%) were included in the experiment. Following incubation, 20 µL Presto blue reagent was added to all the wells, and the plates were further incubated for an additional 3 h. The cell viability was determined by measuring the fluorescence at an excitation of 560 nm and emission of 590 nm using a Perkin Elmer VICTOR Nivo microplate reader^33^. The concentration where 50% of the cell viability was inhibited was calculated by normalising the data to the untreated cell control.

### Masson Fontana assay

The cells were cultured as previously described. The silver nitrate solution was prepared, as described by Kwon-Chung et al.^34^. The cells were fixed and treated with the AgNO_3_ working solution for 40 min at 56 °C (in the dark). Following incubation, the cells were treated with sodium thiosulphate (52.63 mg/mL) for 1 min and rinsed with water. The cells were counterstained using haematoxylin. The washed cells were dehydrated and fixed on microscope slides. Light microscopy images were taken using a Zeiss Primo Vert light microscope and analysed with ImageJ^34^.

### Statistical analysis

All samples were tested in triplicate in three independent experiments unless otherwise stated. The IC_50_ and EC_50_ values, One-way Analysis of Variance (ANOVA), the post-hoc statistical analyses (Tukey’s multiple comparison tests), Pearson correlations, and the Michaelis Menten constants were calculated using GraphPad Prism 5 software. Schrödinger's Canvas was used for the statistical analysis of the QSAR models.

## Supplementary Information

Supplementary Information
