# Supplementary material for: The effect of Aspalathus linearis (Burm.f.) R.Dahlgren and its compounds on tyrosinase and melanogenesis
Source: Sci Rep. 2021 Mar 29;11:7020. doi: 10.1038/s41598-021-86410-z (PMC8007633; doi:10.1038/s41598-021-86410-z)
Supplement: Supplementary file 1 — Supplementary Information [file 41598_2021_86410_MOESM1_ESM.pdf]

SUPPORTING INFORMATION:

The Effect of *Aspalathus linearis* (Burm.f.) R.Dahlgren and its Compounds on Tyrosinase and Melanogenesis

Analike Blom van Staden,[†] *Carel B. Oosthuizen,*[†] *Namrita Lall,*[*,†,‡,§]

[†]Department of Plant and Soil Sciences, University of Pretoria, Pretoria, South Africa

[‡]School of Natural Resources, University of Missouri, USA

[§]College of Pharmacy, JSS Academy of Higher Education and Research, India

SI – Table of content

S1 - Quantification of Pure Compounds Using Ultra-Performance Liquid Chromatography – Quantitative Time of Flight 3

S2 - Tyrosinase Assay 5

S3 – Metal Analysis 7

S4 - Computer modelling 8

S5 - Pearson correlation 20

S6 - Quantitative Structure–Activity Relationship Model 21

S7 - Cytotoxicity Assay 26

S8 - Masson Fontana Assay 27

S1 - Quantification of Pure Compounds Using Ultra-Performance Liquid Chromatography – Quantitative Time of Flight

The spectral data and spectra regarding the Ultra-Performance Liquid Chromatography – Quantitative Time of Flight analysis is provided in Table S 1, and Figure S 1 and S 2.

Table S 1. Spectral data of the major secondary chemical compounds present in the ethanolic extract of unfermented *Aspalathus linearis* (Burm.f.) R.Dahlgren and its fractions quantified using Ultra-Performance Liquid Chromatography – Quantitative Time of Flight

Chemical compounds	Mode	Calibration curve	R^2	LOD[a] (ppm)	LOQ[b] (ppm)
Aspalathin	Positive	y = 26.88x	0.999	< 0.25	< 2.5
Caffeic acid	Negative	y = 112.22x - 7.66	0.999	< 0.25	< 2.5
Catechin	Negative	y = 15.02x - 10.97	0.999	< 0.25	< 2.5
Cinnamic acid	Positive	y = 0.79x + 2.02	0.997	< 0.25	< 2.5
(-)-Epicatechin	Negative	y = 26.56x - 19.68	0.999	< 0.25	< 0.25
Ferulic acid	Negative	y = 31.39x + 0.75	0.994	< 0.25	< 2.5
4-hydroxy benzoic acid	Negative	y = 26.00x + 0.13	0.999	< 25	< 25
Isoquercitrin	Negative	y = 95.22x - 50.05	0.999	< 25	< 25
Luteolin	Positive	y = 0.0545x + 0.23	0.999	< 0.25	< 2.5
Orientin	Positive	y = 0.03x + 0.26	0.981	< 0.25	< 2.5
p-Coumaric acid	Negative	y = 106.42x - 3.15	0.993	< 0.25	< 2.5
n-Propyl gallate	Negative	y = 336.83x - 56.28	0.992	< 0.25	< 0.25
Quercetin	Positive	y = 93.33x - 90.54	0.995	< 2.5	< 2.5
Rosmarinic acid	Negative	y = 63.19x - 8.43	0.995	< 25	< 25
Syringic acid	Negative	y = 25.24x - 3.36	0.994	< 0.25	< 2.5
Vanillic acid	Negative	y = 1.55x - 0.003	0.999	< 0.25	< 2.5
Vitexin	Positive	y = 195.37x -175.73	0.996	< 0.025	< 0.25

[a] Limit of Detection, [b] Limit of quantification.

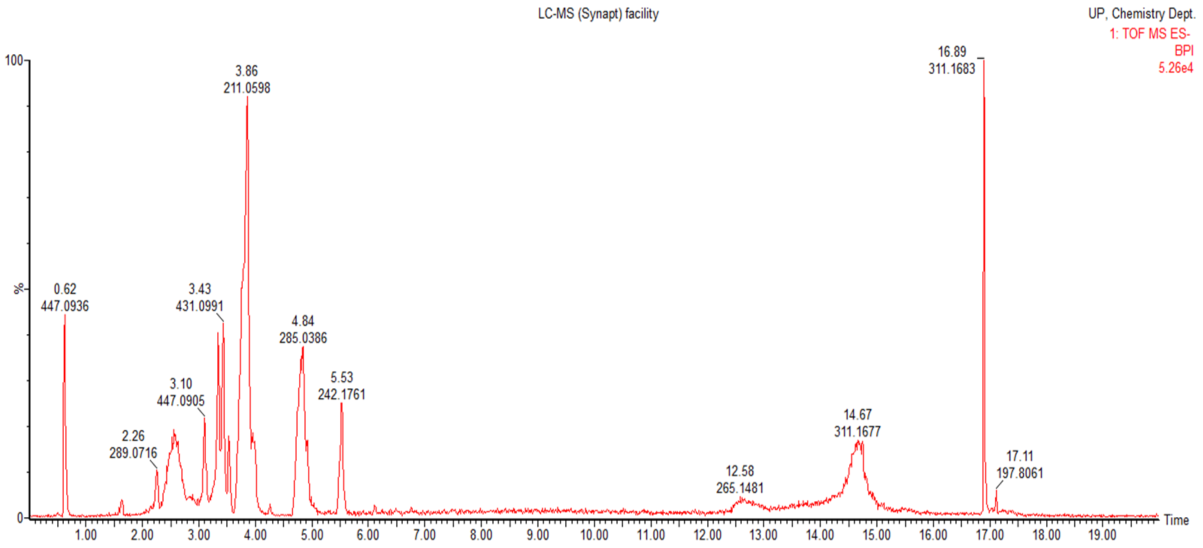

Figure S 1: UPLC-QTOF spectra in negative mode of the ethanolic extract of *Aspalathus linearis* (Burm.f.) R.Dahlgren

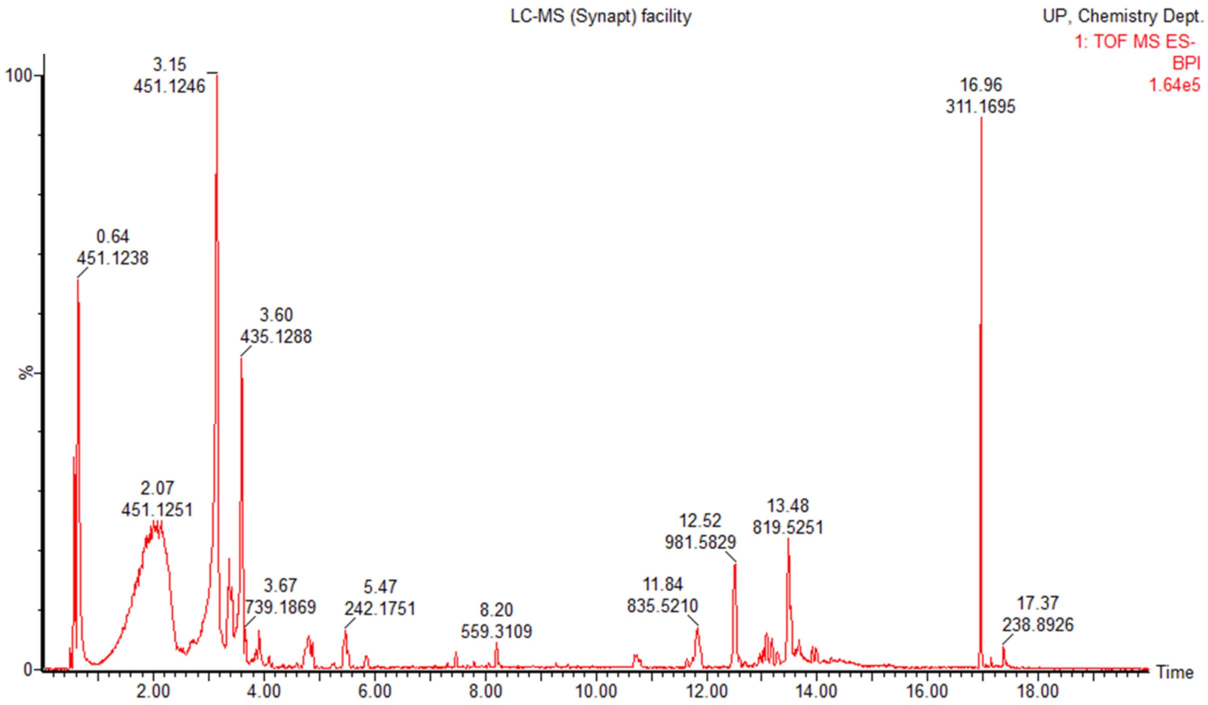

Figure S 2: UPLC-QTOF spectra in positive mode of the ethanolic extract of *Aspalathus linearis* (Burm.f.) R.Dahlgren

S2 - Tyrosinase Assay

The data for the tyrosinase kinetic studies is provided in Figure S 3 to S 5.

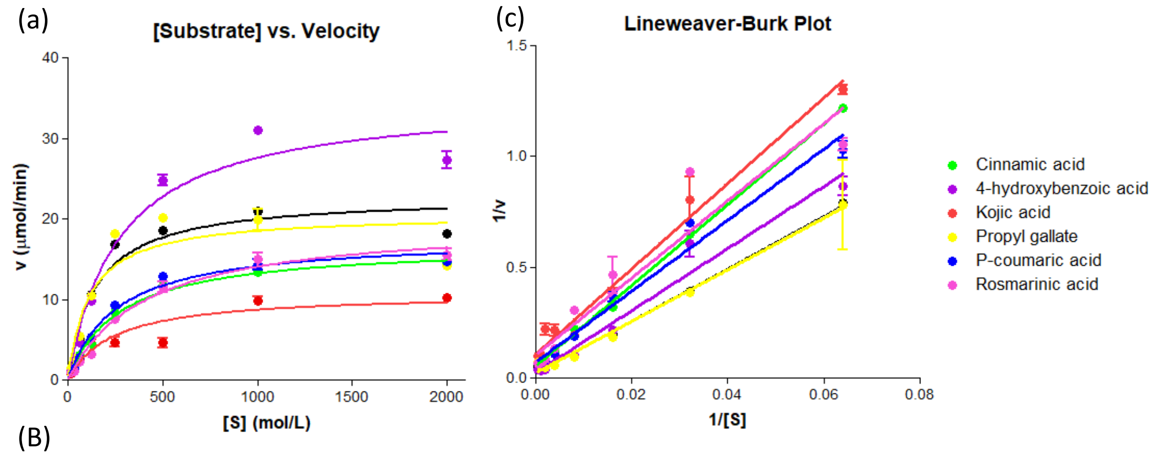

(B)

	v (umol/min)	Cinnamic acid	4-Hydroxybenzoic acid	Kojic acid	Propyl gallate	p-Coumaric acid	Rosmarinic acid
VMAX	22.82 ± 1.07	16.82 ± 0.37	35.00 ± 1.84	10.73 ± 1.01	20.56 ± 1.72	17.71 ± 0.66	19.72 ± 1.01
KM	143.8 ± 25.43	269.9 ± 19.51	267.2 ± 46.26	232.1 ± 64.97	110.80 ± 33.67	254.00 ± 29.91	401.10 ± 57.86

Figure S 3: Enzyme kinetics, namely (a) Michaelis-Menten Plot, (b) Michaelis-Menten Plot data and (c) Lineweaver-Burk Plot of the inhibitors present in *Aspalathus linearis* (Burm.f.) R.Dahlgren (https://www.graphpad.com; GraphPad Prism 5).

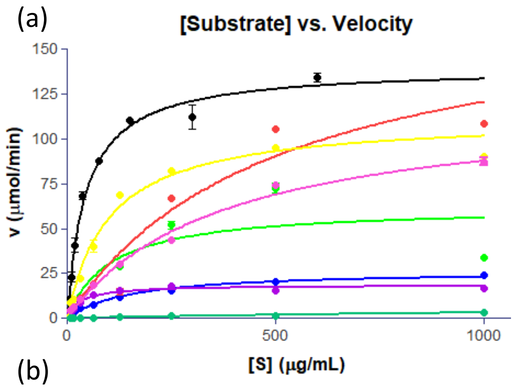

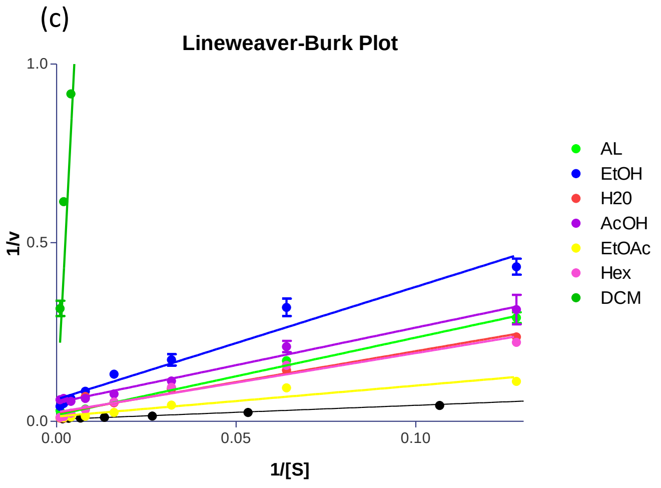

(b)

	VMAX	KM
V (µmol/min)	138.3	42.78
A. linearis EtOH Extract (AL)	61.86	104.0
EtOH Fraction	26.47	149.6
H_2O Fraction	181.3	500.7
AcOH	18.37	31.79
EtAc	111.8	99.75
Hex	120.4	371.4
DCM	11.68	2732

Figure S 4: Enzyme kinetics, namely (a) Michaelis-Menten Plot, (b) Lineweaver-Burk Plot of the ethanolic extract of *Aspalathus linearis* (Burm.f.) R.Dahlgren and its major fractions (https://www.graphpad.com; GraphPad Prism 5).

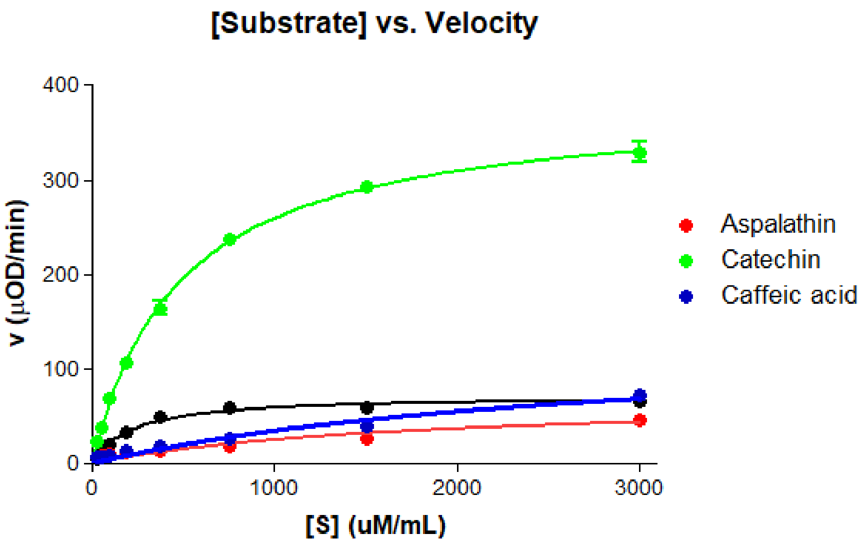

	v (umol/min)	Aspalathin	Catechin	Caffeic acid
VMAX	71.42 ± 2.61	68.73 ± 13.47	382.4 ± 7.03	120.9 ± 15.53
KM	211.5 ± 27.60	1733 ± 660.6	471.0 ± 26.43	2428 ± 553.6

Figure S 5: Michaelis-Menten Plot of the subversive substrates found in the ethanolic extract of *Aspalathus linearis* (Burm.f.) R.Dahlgren. The curve represents the rate of the tyrosinase enzyme in the absence of the substrate L-dopa (https://www.graphpad.com; GraphPad Prism 5).

S3 – Metal Analysis

The metal analysis report obtained from Merieux laboratory is provided in Figure S 6.

MERIEUX NutriSciences
CHELAB S.R.L.

ilac-MRA   ACCREDIA L'ENTE ITALIANO DI ACCREDITAMENTO

LAB N° 0051

TEST REPORT N. 19/000002120

date of issue 03/01/2019

Customer ID 0068079/001

Messrs
SWIFT SILLIKER (PTY) LTD
7 WARRINGTON RD
CLAREMONT
7708
ZA

Sample information

Acceptance number	18.654338.0002
Delivered by	Fedex on 27/12/2018
Receiving Date	27/12/2018
Place of origin	SWIFT SILLIKER (PTY) LTD 7 WARRINGTON RD CLAREMONT 7708 ZA
Sample Description	SSGT 2398 AL UNIVERSITY OF PRETORIA

Sampling information

Sampled by Customer

ANALYTICAL RESULTS

	Value/Uncertain	Unit of measure	LoQ	LoD	Start/end date of analysis	Op. units	Row
**ON SAMPLE AS IT IS**							1
ARSENIC<br>Met.: MP 1288 rev 14 2018	n.d.	mg/kg	0,010	0,0067	31/12/2018--03/01/2019	02	2
CADMIUM<br>Met.: MP 1288 rev 14 2018	n.d.	mg/kg	0,0050	0,0017	31/12/2018--03/01/2019	02	3
MERCURY<br>Met.: MP 1288 rev 14 2018	n.d.	mg/kg	0,0050	0,0017	31/12/2018--03/01/2019	02	4
LEAD<br>Met.: MP 1288 rev 14 2018	n.d.	mg/kg	0,0050	0,0017	31/12/2018--03/01/2019	02	5
COPPER<br>Met.: MP 1288 rev 14 2018	0,060±0,034	mg/kg	0,050	0,017	31/12/2018--03/01/2019	02	6

Operative units

Unit 02 : Via Castellana Resana (TV)

Chemical responsible	Laboratory manager

- The line marked by a star (*) is not accredited by Accredia, member of MLA. - If not otherwise specified, the uncertainty is extended and has been calculated with a coverage factor k=2 corresponding to a probability interval of about 95%. - LoD is the detection limit and identifies a confidence interval of zero with a probabilty interval of about 99%. - LoQ is the limit of quantification."n.d" is not detected and indicates a value inferior to the LoD. "traces (X)" means a value between LoD and LoQ, this value is indicative. "<x" or ">x" indicate inferior or superior to the measurement field of the test. - If not differently specified, the sums are calculated by lower bound criteria (L.B.). - Registration with the number 7 of the Regional List of the laboratories of the Regione Veneto which perform analyses as regards the procedures for the food safety in food industries, as reported in Annex A of DDR n°73 of 16th January 2008

Template 716/SQ rev. 8 Page 2 of 2

Report digitally signed in accordance with Legislative Decree No.82 of March, the 7th, 2005 and s.m.i

The results contained in this Test Report refer only to the analyzed sample. The test report shall not be reproduced except in full, without written approval of Chelab laboratory.

CHELAB S.r.l. Socio Unico, Company subject to the direction and coordination of Mérieux NutriSciences Corporation
Head office: Via Fratta 25 31023 Resana, Italy Phone. + 39 0423.7177 / Fax + 39 0423.715058 www.merieuxnutrisciences.it
VAT nr. 01500900269, R.E.A Treviso n. 156079 Fully paid up € 103.480,00.

Figure S 6: Metal analysis report of the ethanolic extract of *Aspalathin linearis* (Burm.f.) R.Dahlgren conducted by Merieux laboratories.

S4 - Computer modelling

The ChemSpider database IDs for the compounds are provided in Table S2. The docking of compounds **1** to **15** and kojic acid is provided in Figure S 7- S 10 for the tyrosinase and tyrosinase protein 1 structures. Please note that only the structures of aspalathin (**1**) and catechin (**3**) are provided in the manuscript.

Table S 2 ChemSpider database IDs for the compounds docked in the 2Y9X and 5M8P protein structures

**Compound**	**ChemSpider ID**
Aspalathin	9457391
Caffeic acid	600426
Catechin	8711
Cinnamic acid	392447
p-Coumaric acid	553148
Ferulic acid	393368
4-Hydroxybenzoic acid	132
Isoquercetrin	4444361
Kojic acid	3708
Luteolin	4444102
n-Propyl gallate	4778
Quercetin	12269344
Rosmarinic acid	4445104
Syringic acid	10289
Vanillic acid	8155
Vitexin	4444098

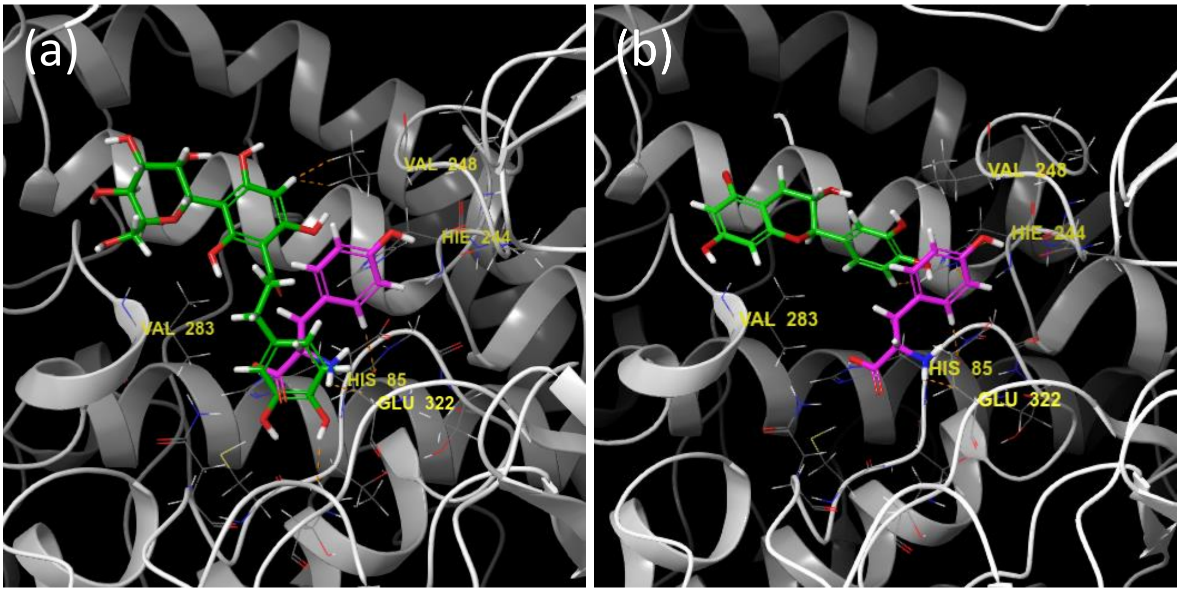

Figure S 7: The docking pose of (a) aspalathin (**1**) and (b) catechin (**2**) was compared to L-tyrosine and showed similar binding and alignment of the core structure to the binding site (https://www.schrodinger.com/maestro; version 12.1.013, release 2019-3).

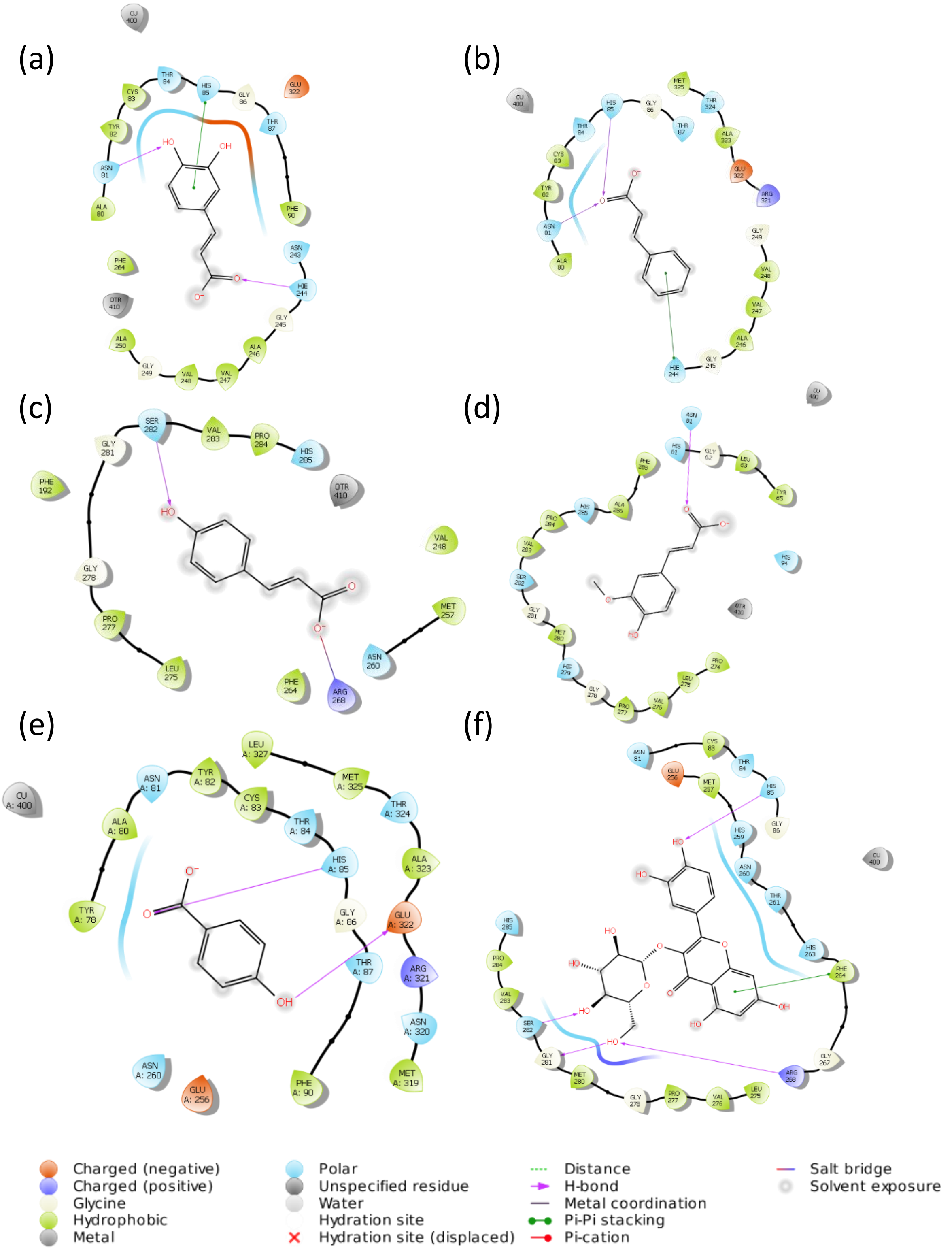

Figure S 8: Two-dimensional protein-ligand interactions of (a) caffeic acid, (b) cinnamic acid, (c) *p*-coumaric acid, (d) ferulic acid, (e) 4-hydroxybenzoic acid and (f) isoquercitrin with the 2Y9X tyrosinase enzyme structure (https://www.schrodinger.com/maestro; version 12.1.013, release 2019-3).

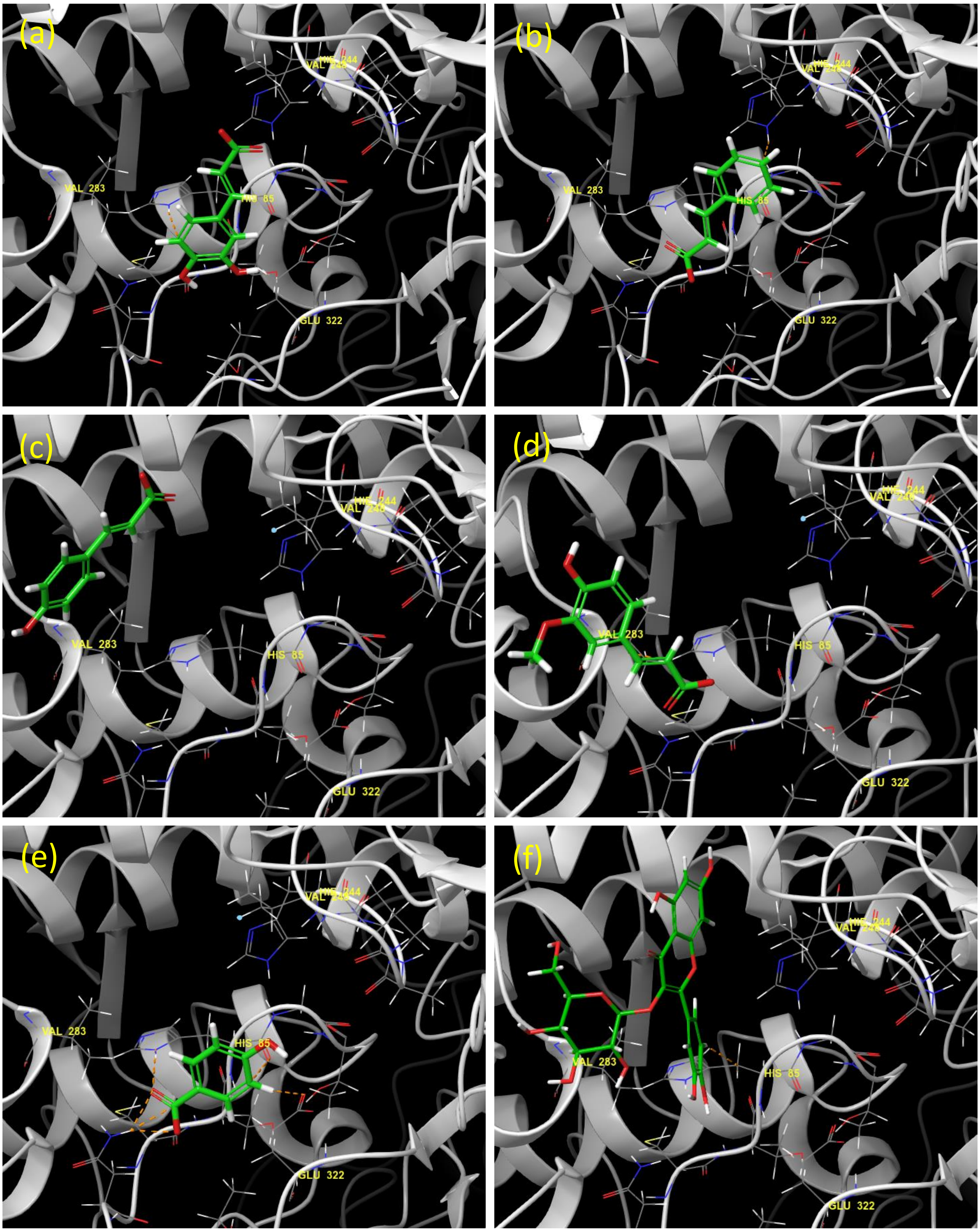

Figure S 9: Three-dimensional protein-ligand interactions of (a) caffeic acid, (b) cinnamic acid, (c) *p*-coumaric acid, (d) ferulic acid, (e) 4-hydroxybenzoic acid and (f) isoquercitrin with the 2Y9X tyrosinase enzyme structure (https://www.schrodinger.com/maestro; version 12.1.013, release 2019-3).

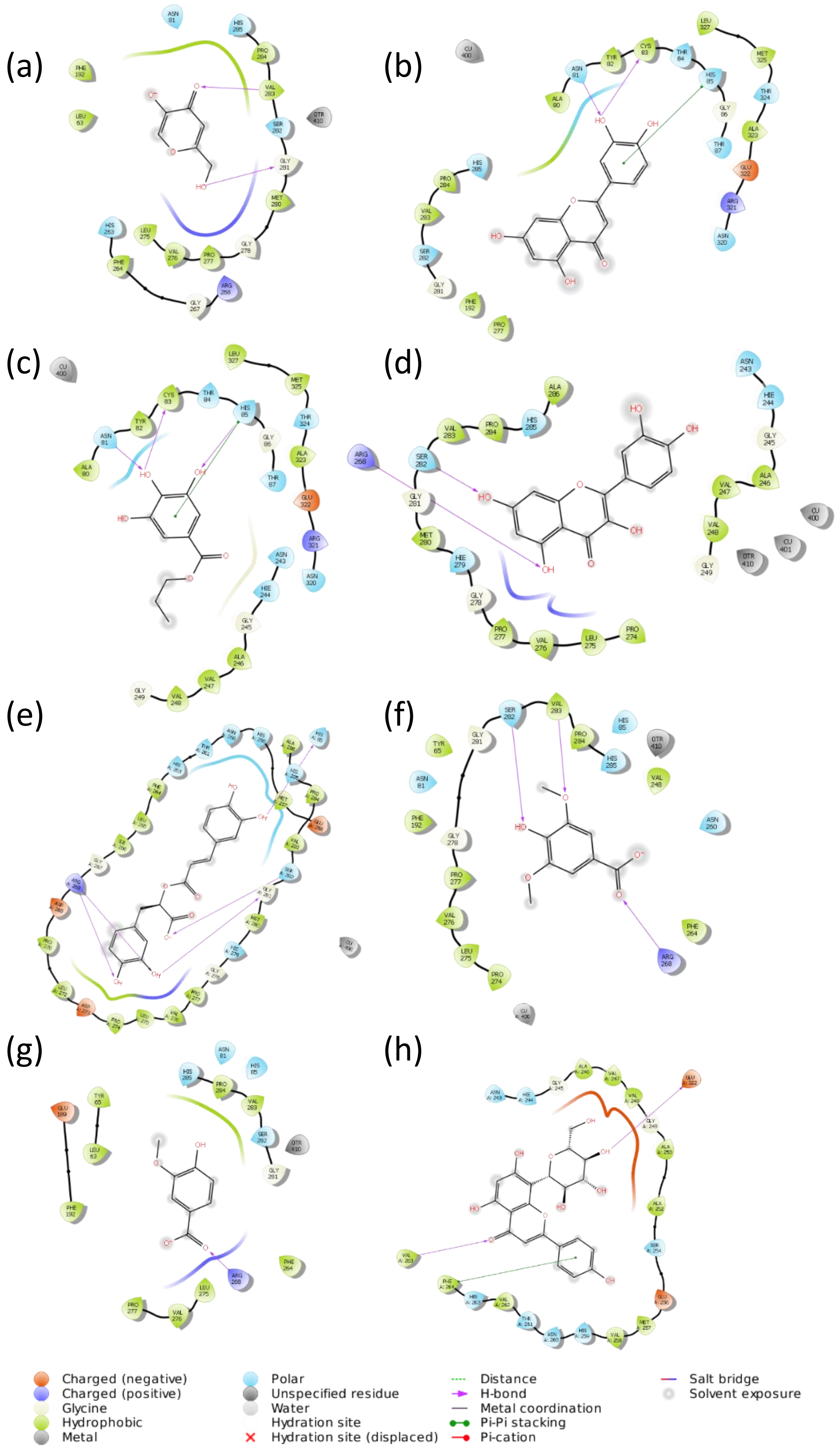

Figure S 10: Two-dimensional protein-ligand interactions of (a) kojic acid, (b) luteolin, (c) *n*-propyl gallate, (d) quercetin, (e) rosmarinic acid, (f) syringic acid, (g) vanillic acid and (h) vitexin with the 2Y9X tyrosinase enzyme structure (https://www.schrodinger.com/maestro; version 12.1.013, release 2019-3).

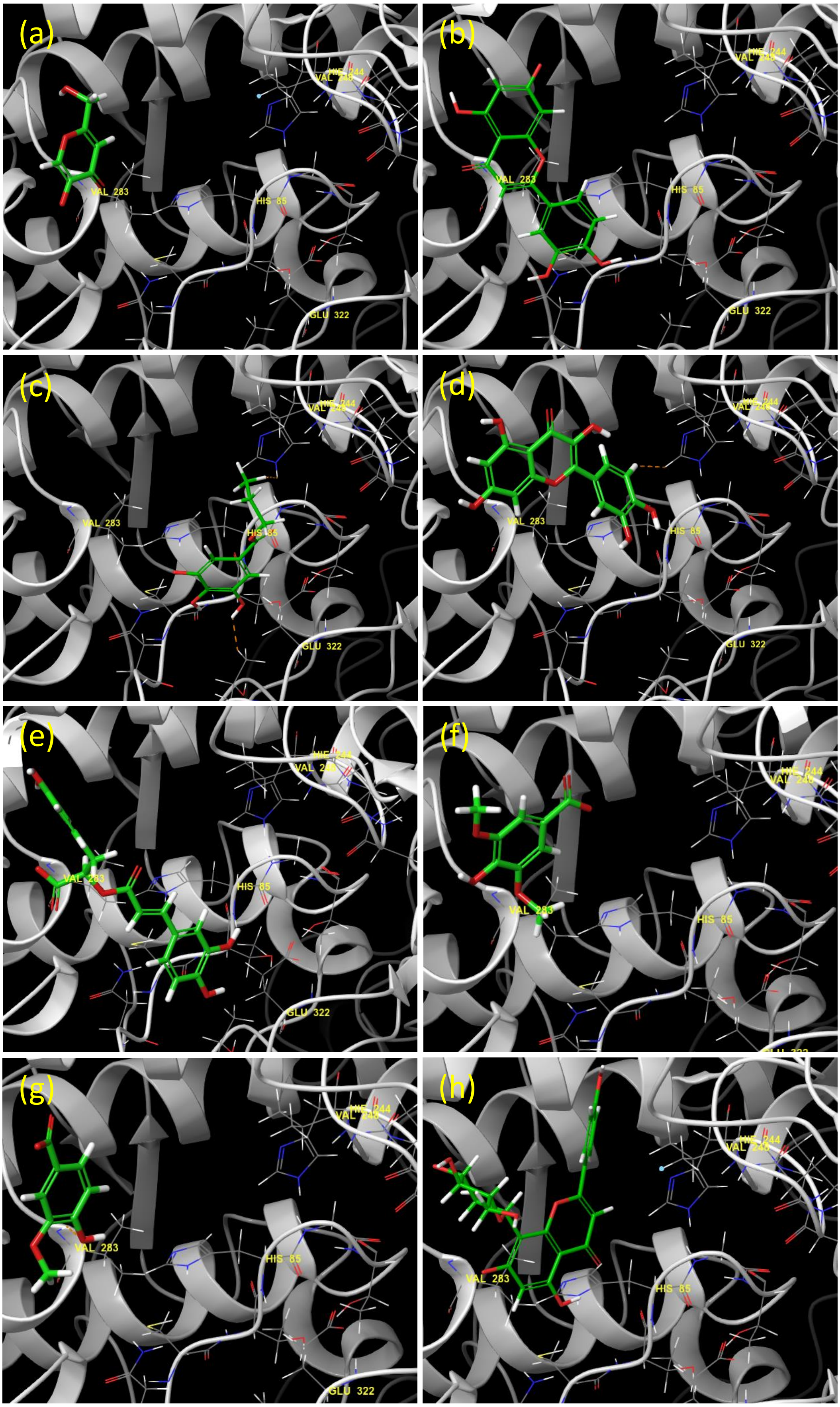

Figure S 11: Three-dimensional protein-ligand interactions of (a) kojic acid, (b) luteolin, (c) *n*-propyl gallate, (d) quercetin, (e) rosmarinic acid, (f) syringic acid, (g) vanillic acid and (h) vitexin with the 2Y9X tyrosinase enzyme structure (https://www.schrodinger.com/maestro; version 12.1.013, release 2019-3).

Table S 3. Hydrogen bond lengths between the compounds and the amino acids at the active site of the tyrosinase structure (PBD: 2Y9X) (https://www.schrodinger.com/maestro; version 12.1.013, release 2019-3).

Compound	Hydrogen bond lengths 2Y9X										
	**HIS 85**	**CYS 83**	**ASN 81**	**SER 282**	**GLY 281**	**ARG 268**	**HIE 244**	**GLU 322**	**VAL 283**	**ARG 250**	**ALA 246**
Aspalathin (**1**)	2.21	1.96	1.99	2.06	1.91	2.19					
Caffeic acid (**2**)			1.92				2.07				
Catechin (**3**)				2.01	2.37		2.41				
Cinnamic acid (**4**)	1.90		2.18								
Ferulic acid (**5**)			2.01								
4-Hydroxybenzoic acid (**6**)	1.81							2.02			
Isoquercitrin (**7**)	2.24			1.94	2.07	2.21					
Luteolin (**8**)		2.23	2.01								
p-Coumaric acid (**9**)				1.90							
n-Propyl gallate (**10**)	2.31	2.09	2.09								
Quercetin (**11**)				1.86		2.13					
Rosmarinic acid (**12**)	2.34			2.06	1.81					2.34	
Syringic acid (**13**)				2.09		2.05			2.10		
Vanillic acid (**14**)						1.63					
Vitexin (**15**)								1.97	2.73		
Kojic acid					1.81				2.22		
L-tyrosine	2.04		2.73					2.52			2.11

Short Long

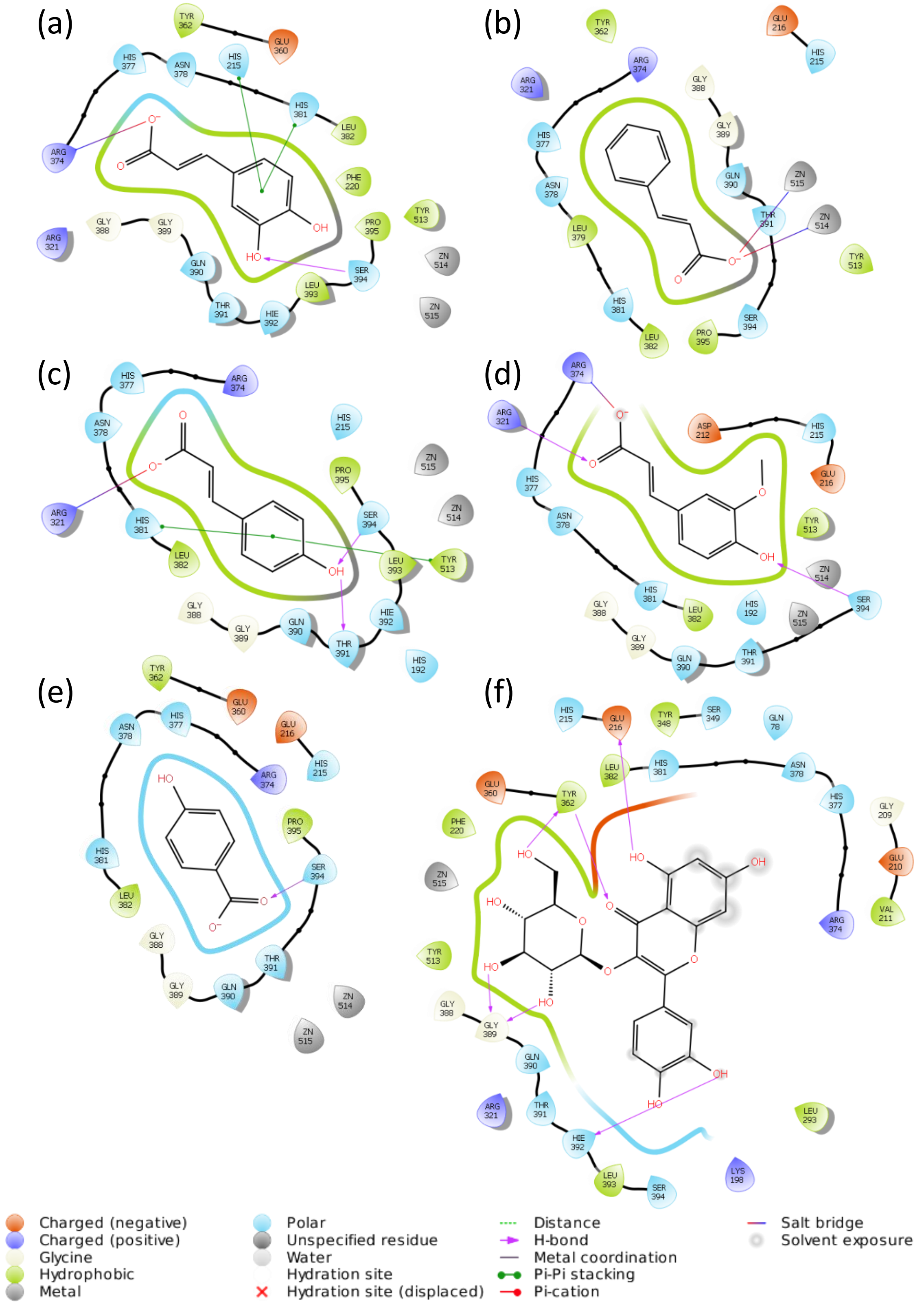

Figure S 12: Two-dimensional protein-ligand interactions of (a) caffeic acid, (b) cinnamic acid, (c) *p*-coumaric acid, (d) ferulic acid, (e) 4-hydroxybenzoic acid and (f) isoquercitrin with the 5M8P tyrosinase protein 1 structure (https://www.schrodinger.com/maestro; version 12.1.013, release 2019-3).

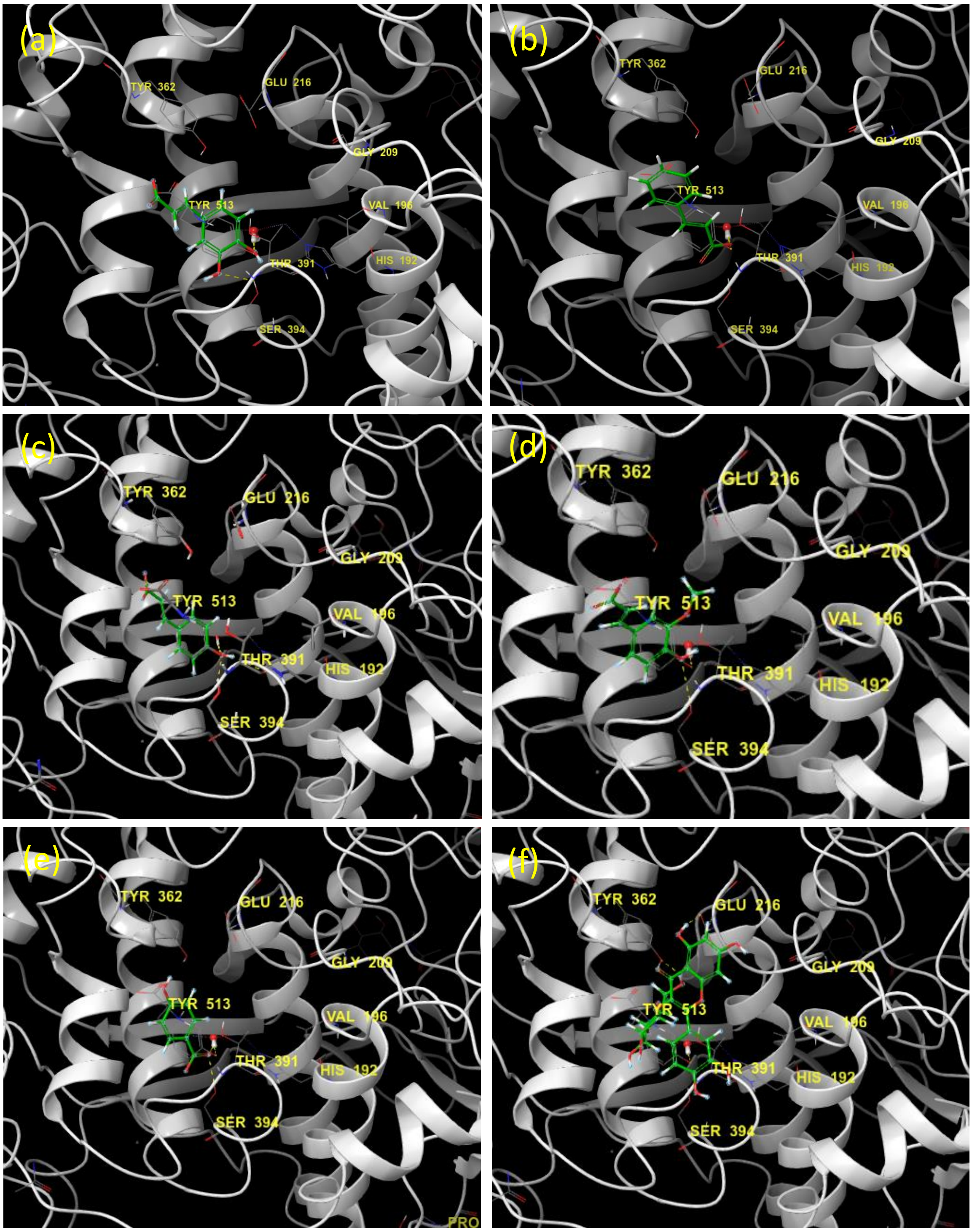

Figure S 13: Three-dimensional protein-ligand interactions of (a) caffeic acid, (b) cinnamic acid, (c) *p*-coumaric acid, (d) ferulic acid, (e) 4-hydroxybenzoic acid and (f) isoquercetrin with the 5M8P tyrosinase protein 1 structure (https://www.schrodinger.com/maestro; version 12.1.013, release 2019-3).

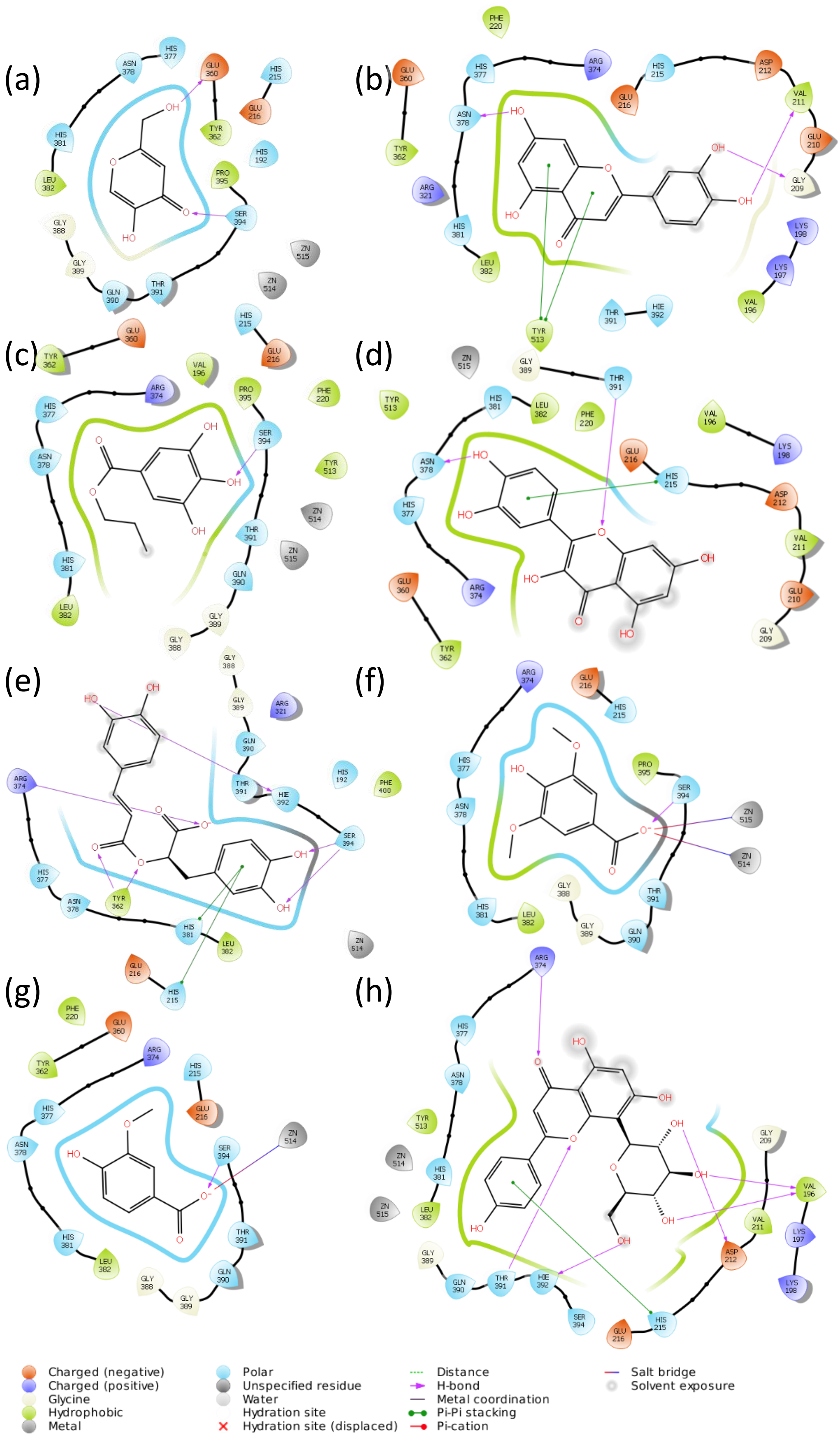

Figure S 14: Two-dimensional protein-ligand interactions of (a) kojic acid, (b) luteolin, (c) *n*-propyl gallate, (d) quercetin, (e) rosmarinic acid, (f) syringic acid, (g) vanillic acid and (h) vitexin with the 5M8P tyrosinase protein 1 structure (https://www.schrodinger.com/maestro; version 12.1.013, release 2019-3).

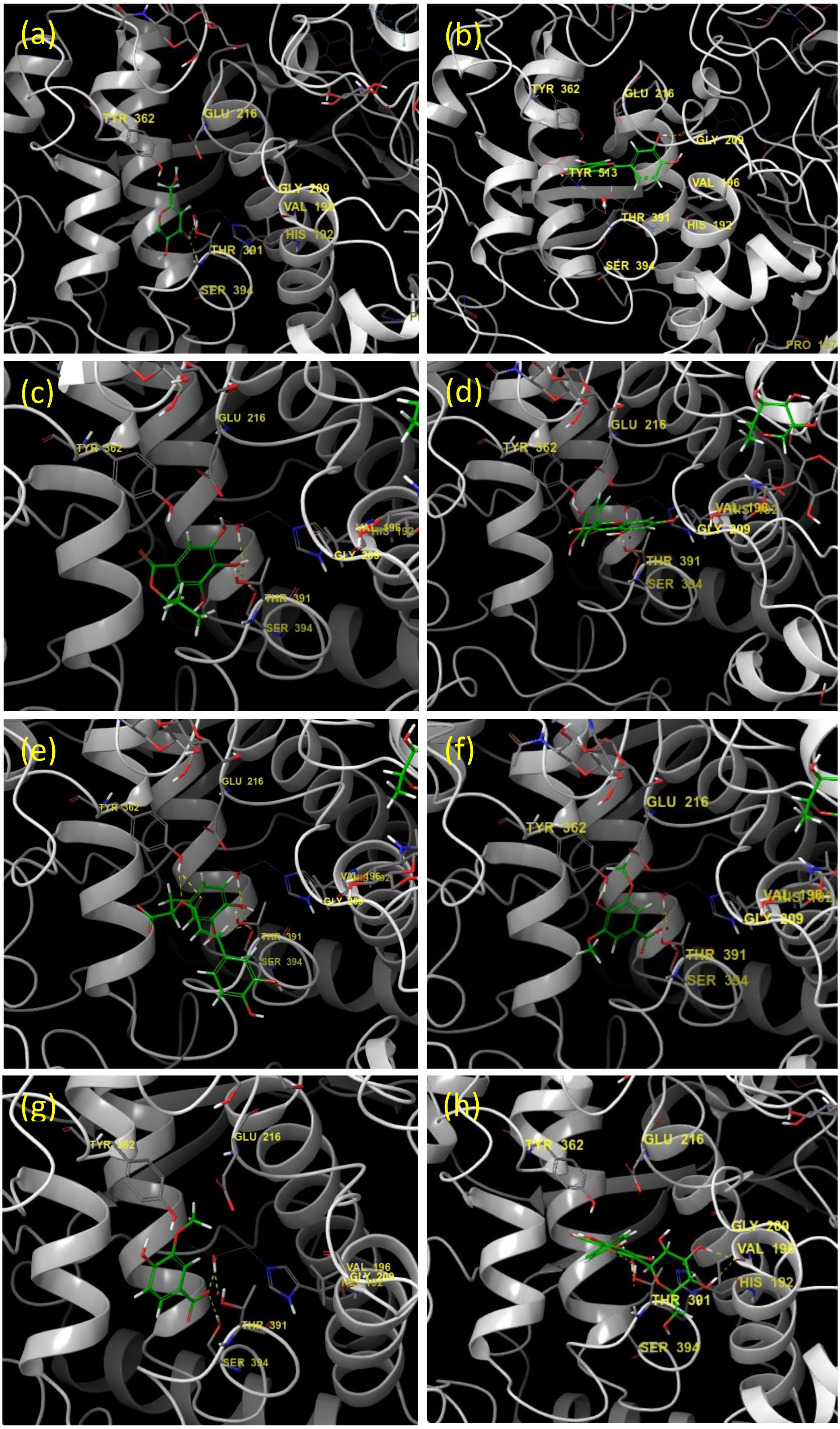

Figure S 15: Three-dimensional protein-ligand interactions of (a) kojic acid, (b) luteolin, (c) *n*-propyl gallate, (d) quercetin, (e) rosmarinic acid, (f) syringic acid, (g) vanillic acid and (h) vitexin with the 5M8P tyrosinase protein 1 structure (https://www.schrodinger.com/maestro; version 12.1.013, release 2019-3).

Table S 4. Hydrogen bond lengths between the compounds and the amino acids at the active site of the tyrosinase-related protein 1 structure (PBD: 5M8P) (https://www.schrodinger.com/maestro; version 12.1.013, release 2019-3).

Compounds	**Hydrogen bond lengths 5M8P**															
	**GLY 209**	**GLU 216**	**TYR 362**	**GLY 388**	**SER 394**	**ASN 378**	**VAL 196**	**ARG 321**	**GLY 389**	**HIE 392**	**VAL 211**	**THR 391**	**ARG 374**	**VAL 196**	**ASP 212**	**GLU 360**
Aspalathin (**1**)	1.95	2.03	2.14	2.42	2.27											
Caffeic acid (**2**)					2.67											
Catechin (**3**)						1.86	1.80									
Cinnamic acid (**4**)																
Ferulic acid (**5**)					2.42			2.33								
4-Hydroxybenzoic acid (**6**)					1.97											
Isoquercitrin (**7**)		1.79	1.79						1.89	2.39						
Luteolin (**8**)	1.91					1.89					2.06					
p-Coumaric acid (**9**)					1.79							2.78				
n-Propyl gallate (**10**)					2.18											
Quercetin (**11**)						2.00						2.11				
Rosmarinic acid (**12**)			2.43		1.94					2.25			1.94			
Syringic acid (**13**)					1.82											
Vanillic acid (**14**)					2.05											
Vitexin (**15**)										2.21		2.51	1.96	1.86	2.38	
Kojic acid					1.96											2.34

Short Long

S5 - Pearson correlation

The data obtained using the Pearson correlation is provided in Table S 5 and Figure S 16.

Table S 5. Pearson correlation, assuming a Gaussian distribution between the tyrosinase activity and the docking scores for the tyrosinase structure (PBD: 2Y9X) and for the tyrosinase related protein 1 structure (PBD: 5M8P) (https://www.graphpad.com; GraphPad Prism 5)

Pearson Correlation			
	**Correlation between Tyrosinase activity and 2Y9X docking score**	**Correlation between Tyrosinase activity and 5M8P docking score**	**Correlation between 2Y9X and 5M8P docking scores**
Number of XY Pairs	16	16	16
Pearson r	-0.5696	-0.4719	0.7618
95% confidence interval	-0.8308 to -0.1028	-0.7842 to 0.03119	0.4275 to 0.9128
P value (two-tailed)	0.0213	0.065	0.0006
P value summary	*	ns	***
Is the correlation significant? (alpha=0.05)	Yes	No	Yes
R squared	0.3244	0.2227	0.5804

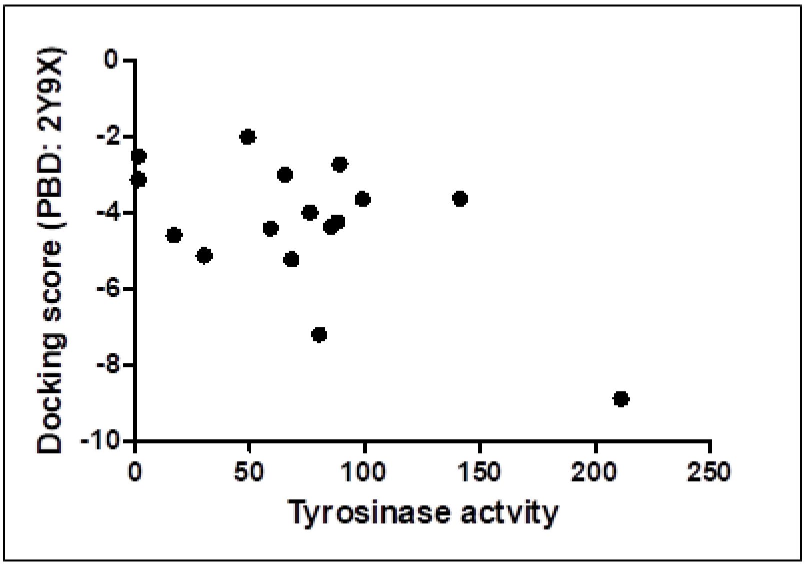

Figure S 16: Pearson correlation, assuming Gaussian distribution, between the tyrosinase activity and the docking scores for the tyrosinase structure (PBD: 2Y9X) (https://www.graphpad.com; GraphPad Prism 5).

S6 - Quantitative Structure–Activity Relationship Model

The data obtained using the Quantitative Structure–Activity Relationship Model is provided in Table S 6 to S 9.

Table S 6. Structure Activity Relationship Table for the compounds identified in the ethanolic extract of *Aspalathus linearis* (Burm.f.) R.Dahlgren (https://www.schrodinger.com/canvas; version 4.1.013, release 2019-3).

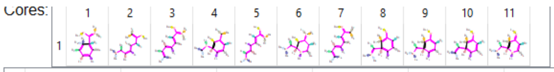

	Input #	Structure Name	R1	R2	R3	R4	R5	R6	R7	R8	R9	R10
1	1	Vanillic acid	HO	O	H	H	Null	Null	Null	H	Null	H
2	2	Quercetin	H	OH	O	O	O	OH OH	O	H	Null	H
3	3	P-Coumaric Acid	H		H	H	H	Null	Null	HO		H
4	4	Syringic acid	Null	OH	Null	O	H	O	OH	H	H	H
5	5	Cinnamic Acid	H		H	H	H	Null	Null		H	H
6	6	Caffeic Acid	H	HO	HO	H	H	Null	Null	H	Null	H
7	7	Aspalathin	H	Null	H		OH	H	Null	H	Null	HO
8	8	Luteolin	H	OH	H	OH O OH	H	HO O OH	HO O HO	HO	HO	Null
9	9	Ferulic Acid	H		O	H	H	Null	Null	HO		H
10	10	Propyl Gallate	Null	H	O	OH	OH	H	Null	Null	Null	H
11	11	(=)-catechin	Null	H	HO OH HO O	H	OH	H	Null	HO OH HO O	Null	H
12	12	Rosmarinic Acid	Null	H	H	H	OH	H	Null		H	H
13	13	Isoquercitrin	H	Null	H		OH	H	Null	H	Null	HO
14	14	4-Hydroxybenz...	HO	H	H	H	Null	Null	Null	H	Null	H
15	15	Vitexin	H	Null	H		OH	H	Null	H	Null	HO
16	16	Protocatechuic...	Null	H	O	H	OH	H	Null	H	Null	H
17	17	Kojic acid	Null	Null	H	H	OH	H	Null	H	Null	Null

Table S 7. The tyrosinase property activity cliffs of the R-group analysis of the compounds identified in the ethanolic extract of *Aspalathus linearis* (Burm.f.) R.Dahlgren (https://www.schrodinger.com/canvas; version 4.1.013, release 2019-3).

	Deviation	Structure 1	Structure 2	Δ Fingerprint	| Δ Property |
1	1.23	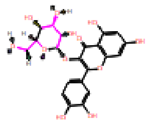 Isoquercitrin	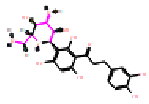 Aspalathin	10.0	131.0
2	-1.19	89.00 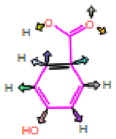 4-Hydroxybenzoic Acid	88.00 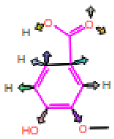 Vanillic acid	5.0	1.0
3	-1.9	68.00 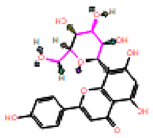 Vitexin	80.00 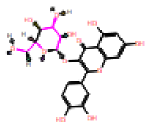 Isoquercitrin	10.0	12.0
4	1.29	68.00 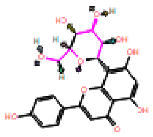 Vitexin	211.00 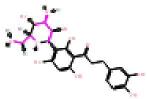 Aspalathin	8.94	143.0
5	-1.16	49.00 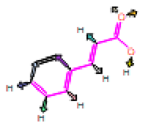 Cinnamic Acid	1.50 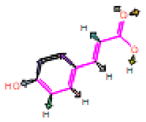 P-Coumaric Acid	4.47	47.5
6	-0.66	65.00 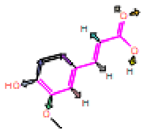 Ferulic Acid	1.50 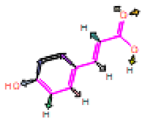 P-Coumaric Acid	5.83	63.5

Deviation:
greater than 4
between 2 and 4
between -2 and 2
between -4 and -2
less than -4

Table S 8. The docking score for the tyrosinase structure (PBD: 2Y9X) property activity cliffs of the R-group analysis of the compounds identified in the ethanolic extract of *Aspalathus linearis* (Burm.f.) R.Dahlgren (https://www.schrodinger.com/canvas; version 4.1.013, release 2019-3).

	Deviation	Structure 1	Structure 2	Δ Fingerprint	| Δ Property |
1	1.39	-7.19 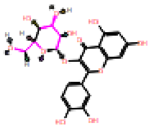 Isoquercitrin	-8.88 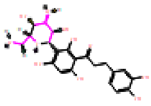 Aspalathin	11.27	1.69
2	-1.21	-2.71 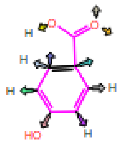 4-Hydroxybenzoic Acid	-4.24 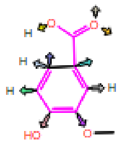 Vanillic acid	5.29	1.53
3	0.99	-5.21 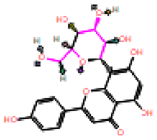 Vitexin	-7.19 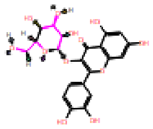 Isoquercitrin	10.86	1.98
4	1.82	-5.21 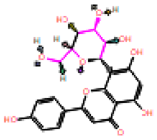 Vitexin	-8.88 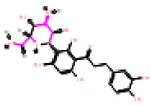 Aspalathin	10.82	3.67
5	-1.15	-2.00 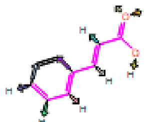 Cinnamic Acid	-2.51 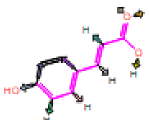 P-Coumaric Acid	4.58	0.51
6	-0.99	-2.99 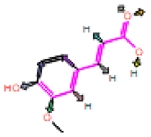	-2.51 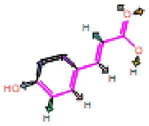	6.0	0.48

Deviation:
greater than 4
between 2 and 4
between -2 and 2
between -4 and -2
less than -4

Table S 9. The docking score for the tyrosinase related protein 1 structure (PBD: 5M8P) property activity cliffs of the R-group analysis of the compounds identified in the ethanolic extract of *Aspalathus linearis* (Burm.f.) R.Dahlgren (https://www.schrodinger.com/canvas; version 4.1.013, release 2019-3).

	Deviation	Structure 1	Structure 2	Δ Fingerprint	| Δ Property |
1	1.19	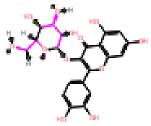 Isoquercitrin	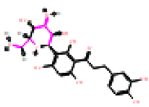 Aspalathin	11.27	200.0
2	1.49	258.10 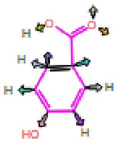 4-Hydroxybenzoic Acid	0.0 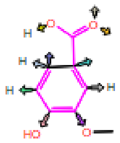 Vanillic acid	5.29	258.1
3	-1.53	200.00 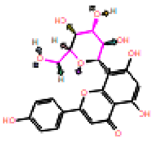 Vitexin	200.00 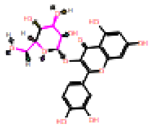 Isoquercitrin	10.86	0.0
4	1.08	200.00 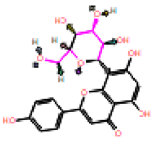 Vitexin	0.0 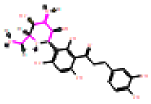 Aspalathin	10.82	200.0
5	-1.14	139.80 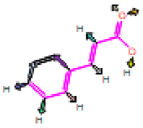 Cinnamic Acid	25.03 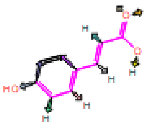 P-Coumaric Acid	4.58	114.77
6	-1.25	0.0 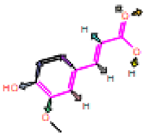 Ferulic Acid	25.03 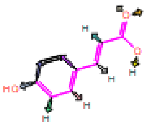 P-Coumaric Acid	6.0	25.03

Deviation:
- greater than 4
- between 2 and 4
- between -2 and 2
- between -4 and -2
- less than -4

S7 - Antiproliferation Assay

The data obtained for the antiproliferation assay is provided in Table S 10.

Table S 10. Sigmoidal dose response curve analysis of the ethanolic extract of *Aspalathus linearis* (Burm.f.) R.Dahlgren (AL), its fractions and the compounds aspalathin and catechin (https://www.graphpad.com; GraphPad Prism 5)

**Sigmoidal dose-response (variable slope)**									
**Best-fit values**	**AL**	**DCM**	**Acetic**	**H_2O**	**EtAc**	**EtOH**	**Hexane**	**Aspalathin**	**Catechin**
Bottom	0	0	0	0	0	0	0	0	0
Top	100	100	100	100	100	100	100	100	100
$LogEC_{50}$	2.22	2.10	2.32	2.56	2.37	2.36	2.09	2.34	2.22
HillSlope	-2.18	-2.67	-1.76	-1.93	-4.45	-2.19	-2.18	-5.86	-3.06
EC_{50}	164.80	126.50	208.50	359.60	231.70	230.70	123.20	217.70	166.20
**Std. Error**									
$LogEC_{50}$	0.05	0.06	0.03	0.06	0.04	0.04	0.07	0.08	0.03
HillSlope	0.48	0.83	0.23	0.53	2.08	0.37	0.67	11.81	0.71
**95% CI (profile likelihood)**									
$LogEC_{50}$	2.12 to 2.32	1.99 to 2.22	2.25 to 2.39	2.44 to 2.704	2.28 to 2.44	2.28 to 2.44	1.95 to 2.24	2.16 to 2.52	2.15 to 2.29
HillSlope	-3.36 to -1.51	-6.36 to -1.69	-2.33 to -1.34	-3.34 to -1.19	6.76 to -2.31	-3.42 to -1.51	-4.33 to -1.34	-32.17 to 20.46	-4.63 to -1.48
EC50	130.30 to 208.80	96.55 to 166.60	177 to 245.50	274.80 to 505.60	188.50 to 275.40	192 to 274	88.26 to 174.40	144.50 to 328.00	140.30 to 196.80
**Goodness of Fit**									
Degrees of Freedom	22	22	22	22	22	22	22	10	10
R square	0.89	0.87	0.92	0.76	0.86	0.90	0.82	0.67	0.89
Absolute Sum of Squares	4313	6943	1671	4735	5569	2349	8685	2130	1203
Sy.x	14.00	17.77	8.71	14.67	15.91	10.33	19.87	14.60	10.97
**Constraints**									
Bottom	0	0	0	0	0	0	0	0	0
Top	100	100	100	100	100	100	100	100	100
Number of points analysed	24	24	24	24	24	24	24	12	12

S8 - Masson Fontana Assay

The data obtained for the Masson Fontana assay is provided in Table S 11.

Table S 11. One-way analysis of variance and the post-hoc statistical analyses (Tukey's multiple comparison tests) of the number of melanin produced in melanocytes (UCT-Mel1) treated with the ethanolic extract of *Aspalathus linearis* (Burm.f.) R.Dahlgren (AL), its fractions and the compounds aspalathin and catechin vs the untreated (negative) melanocytes (https://www.graphpad.com; GraphPad Prism 5)

**One-way analysis of variance**					
P value	P<0.0001				
P value summary	***				
Are means signif. different? (P < 0.05)	Yes				
Number of groups	11				
F	7.028				
R squared	0.5273				
**ANOVA Table**	**SS**	**df**	**MS**		
Treatment (between columns)	1511	10	151.1		
Residual (within columns)	1355	63	21.5		
Total	2866	73			

**Dunnett's Multiple Comparison Test**	**Mean Diff.**	**q**	**Significant? P < 0.05?**	**Summary**	**95% CI of diff**
Negative vs aMSH 4.5uM	-7.413	2.841	Yes	*	-14.71 to -0.1129
Negative vs aMSH 9uM	-9.156	4.51	Yes	***	-14.83 to -3.476
Negative vs aMSH 18uM	-15.06	5.771	Yes	***	-22.36 to -7.760
Negative vs AL	-9.23	4.875	Yes	***	-14.53 to -3.934
Negative vs AcOH	-9.572	4.273	Yes	***	-15.84 to -3.306
Negative vs EtOAc	-9.107	3.49	Yes	**	-16.41 to -1.807
Negative vs EtOH	-11.17	4.663	Yes	***	-17.86 to -4.467
Negative vs H_2O	-8.718	4.107	Yes	**	-14.66 to -2.780
Negative vs Aspalathin	-8.382	3.742	Yes	**	-14.65 to -2.116
Negative vs Catechin	-15.08	6.297	Yes	***	-21.78 to -8.380
